# Supplementary figures and images for: A computational approach to identify phytochemicals as potential inhibitor of acetylcholinesterase: Molecular docking, ADME profiling and molecular dynamics simulations
Source: PLoS One. 2024 Jun 4;19(6):e0304490. doi: 10.1371/journal.pone.0304490 (PMC11149856; doi:10.1371/journal.pone.0304490)

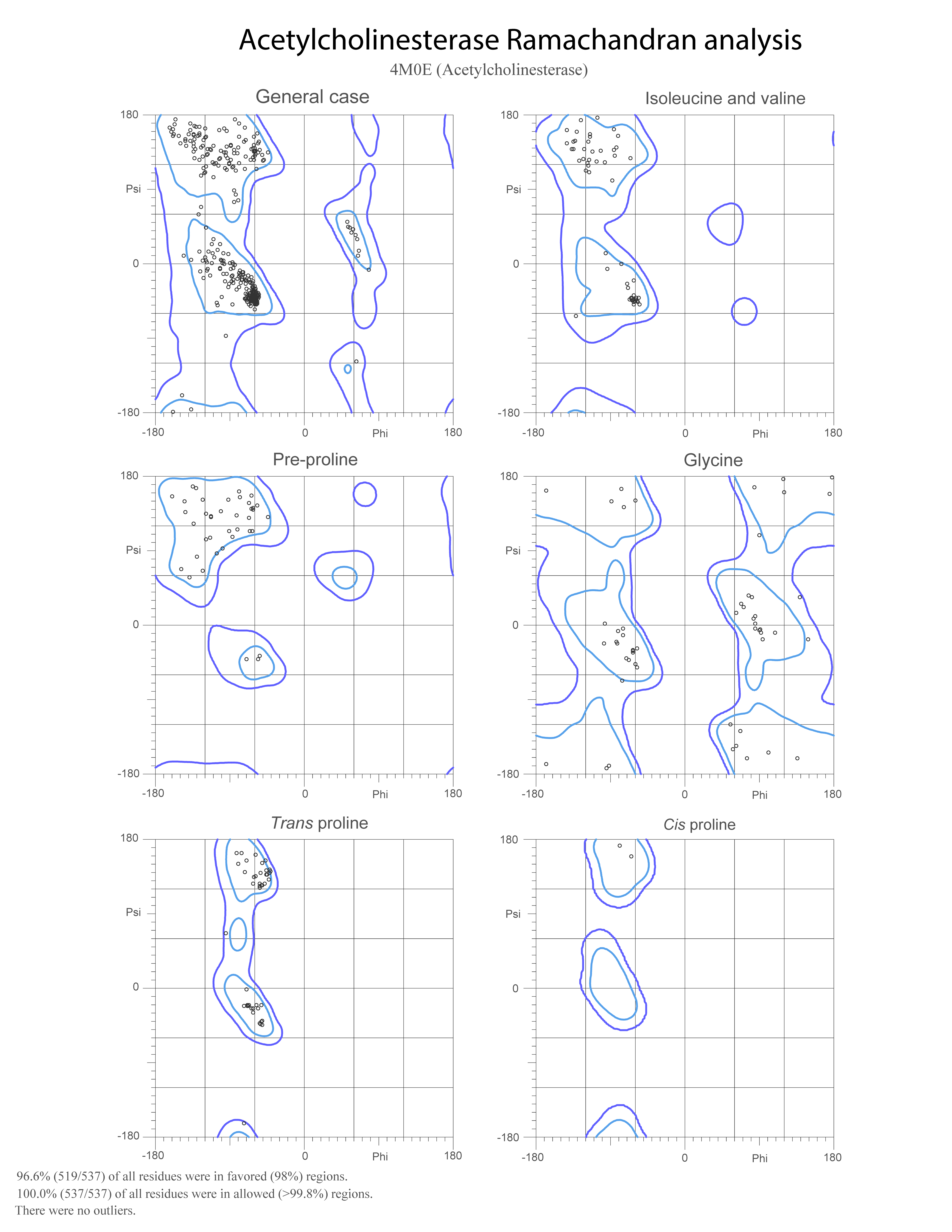

Supplement: S1 Fig — The Ramachandran plot shows the statistical distribution of the combinations of the backbone dihedral angles ϕ and ψ. In theory, the allowed regions of the Ramachandran plot show which values of the Phi/Psi angles are possible for an amino acid, X, in an ala-X-ala tripeptide (Wiltgen, 2019). The Ramachandran plot analysis of protein AChE showed high conformational quality, with no outliers identified. All 537 residues (100%) were in acceptable regions (>99.8%), with 96.6% (519/537) falling within favoured regions (>98%). (TIF) [file pone.0304490.s001.tif]

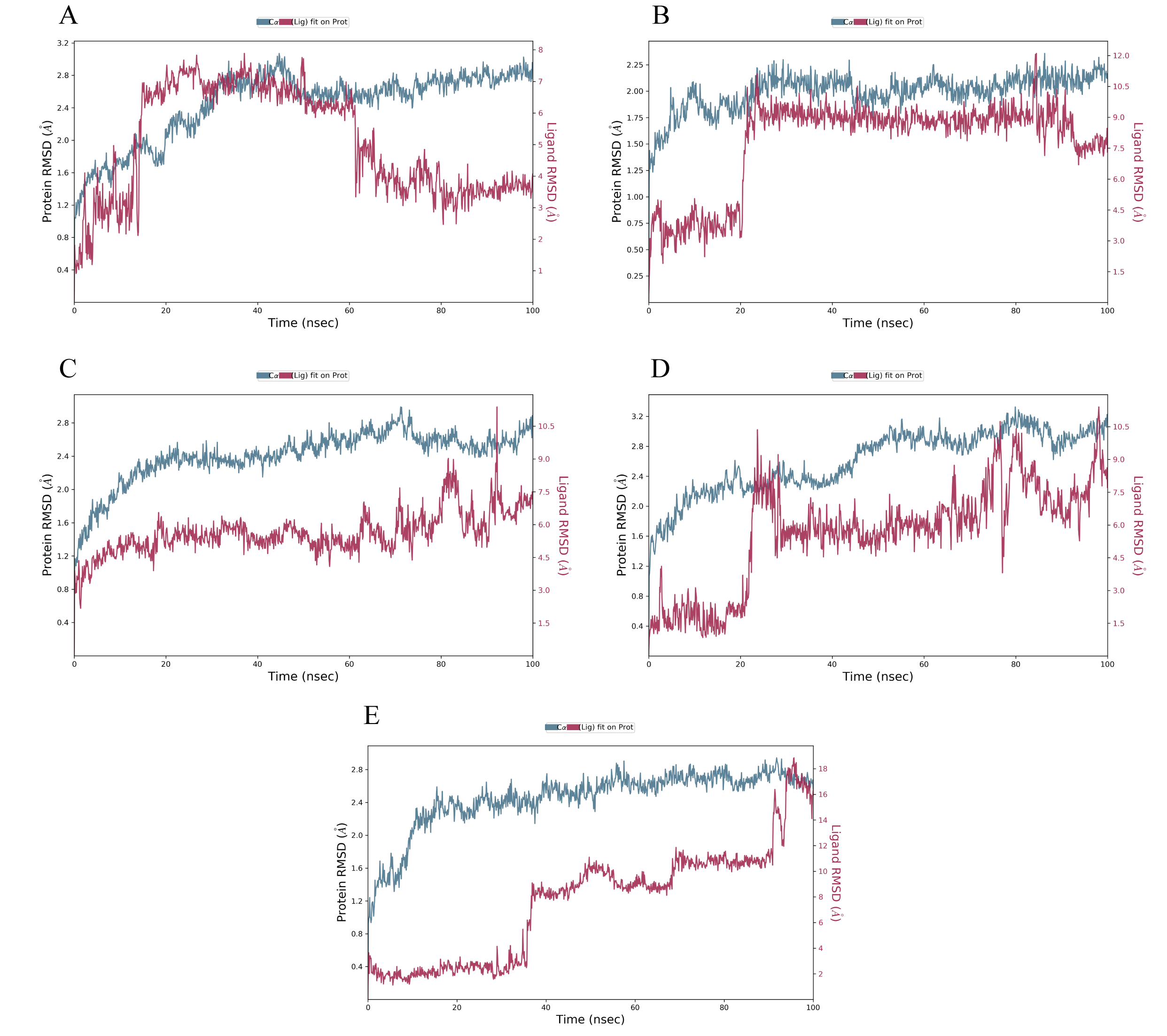

Supplement: S2 Fig — Results of rest 5 complexes. Complexes 2, 4, 5, 7 and 8 are subjected to a 100-nanosecond molecular dynamics simulation using the Desmond software. A) RMSD of Complex_2, B) RMSD of Complex_4, C) RMSD of Complex_5, D) RMSD of Complex_7, E) RMSD of Complex_8. The root means square deviation (RMSD) between the ligand and protein exhibits temporal constancy, thereby can’t able tov ensure stability. Nevertheless, complex_4 demonstrated persistent stability over 90 ns simulation but rest of 10 ns it deviated, and ligand don’t fit to the protein, suggesting that the interaction between the protein and ligand remains intact throughout the 90 ns duration but ultimate result is not good all over the time. The rest of the complexes don’t show any stable interaction throughout the simulation. It may cause of ion imbalance of the binding pore. Overall binding interaction is not favorable, and further investigation is required for the other parameters. (TIF) [file pone.0304490.s002.tif]

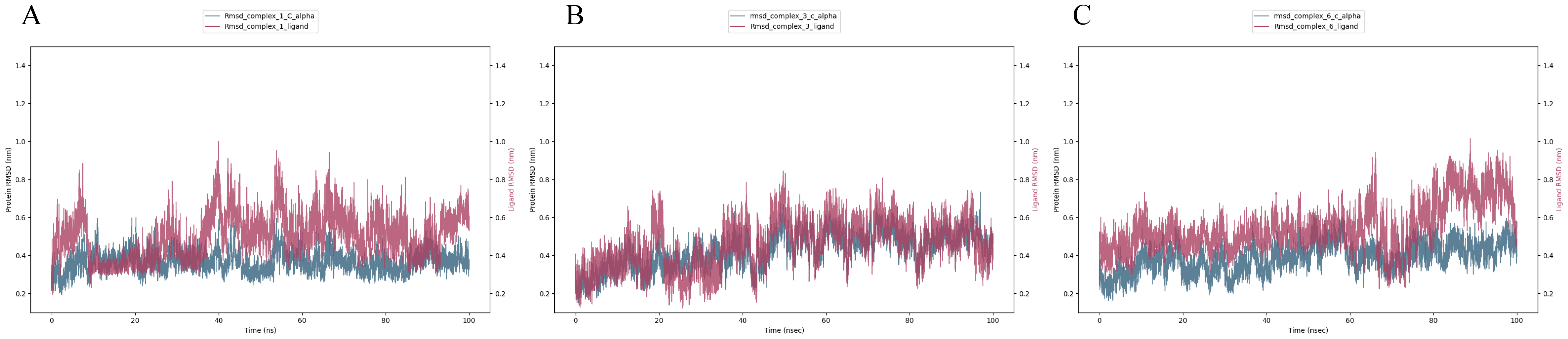

Supplement: S3 Fig — Cross-validation of the stability by another simulation machine GROMACS. In the graph, green, and red colour represented protein carbon alpha chains, ligand respectively. A) Complex_1 showed steady protein RMSD, and ligands overlapped only for a short time. B) Complex_3 displayed increasing RMSD and deemed well synchronized with the ligand RMSD. C) Complex_6 demonstrates overlapping RMSD of protein and ligand till 60 ns afterward ligand RMSD goes higher. Overall, RMSD of the complexes ensures stability and compactness although further validation may need. Nevertheless, the overall binding interaction is not significantly unfavourable, and further investigation is required for the other parameters. (TIF) [file pone.0304490.s003.tif]

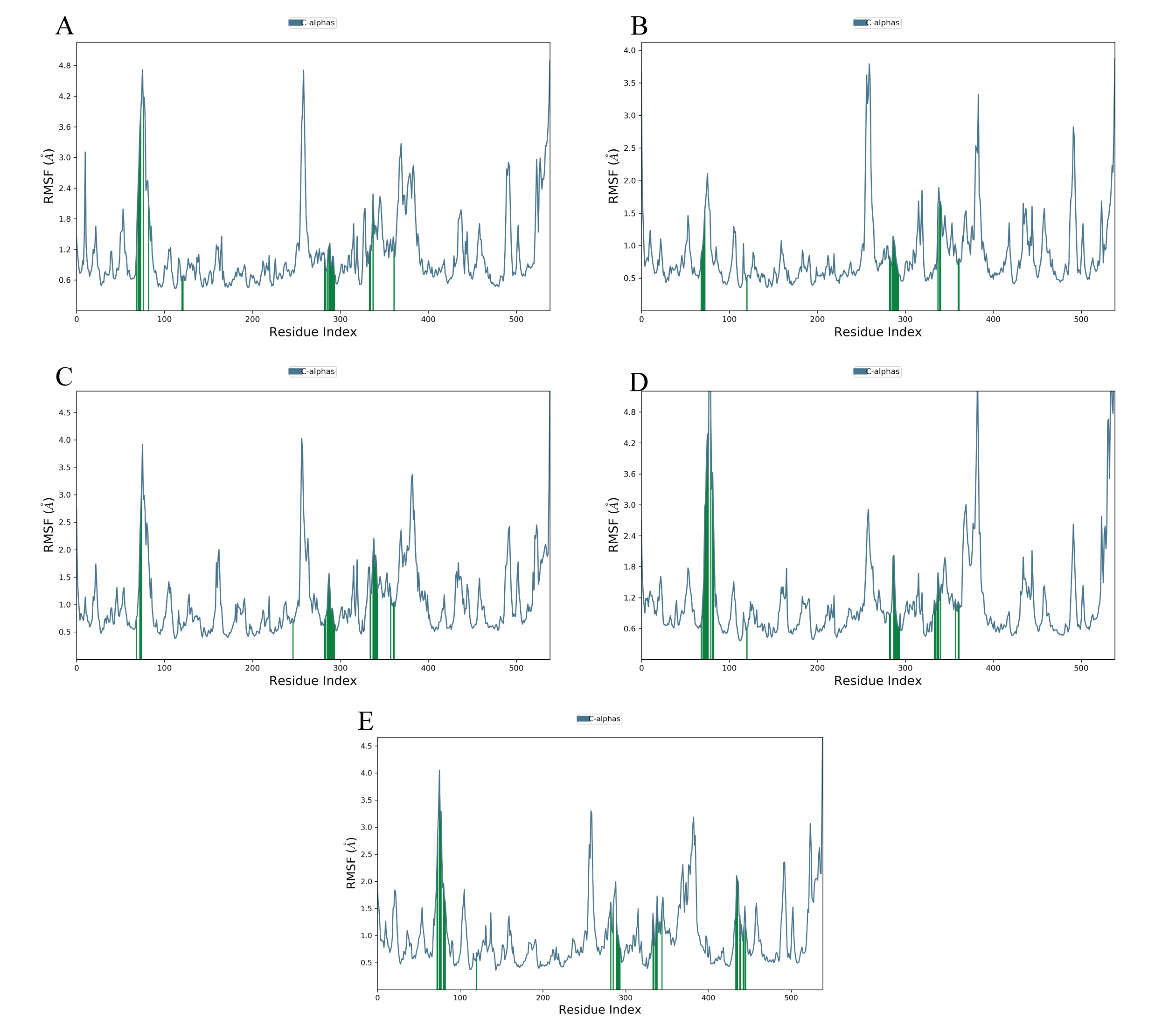

Supplement: S4 Fig — A- Root Mean Square Fluctuation (RMSF) of Complex_2, B- RMSF of Complex_4, C- RMSF of Complex_5, D- RMSF of Complex_7, E- RMSF of Complex_8. The interpretation of the results is justified by Several significant fluctuations. The fluctuation primarily arises when the ligand interacts with the protein residues. Complex_7 has shown two major fluctuations as 180 residues and 395 residues. Overall, others exhibit significant fluctuations on the green vertical bar, which signify the hydrogen bond contact between the ligand molecule and the protein. (TIF) [file pone.0304490.s004.tif]

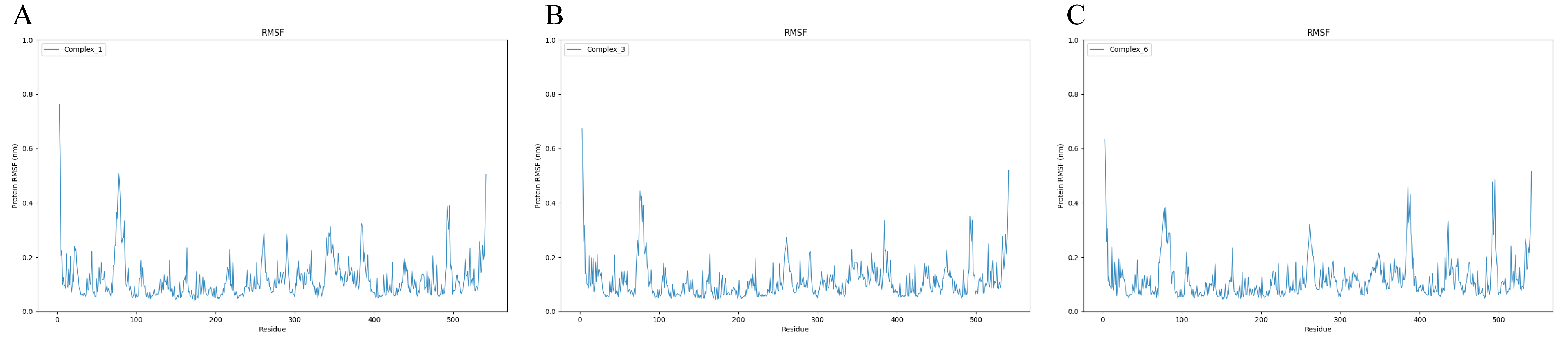

Supplement: S5 Fig — A- Root Mean Square Fluctuation (RMSF) of Complex_1, B- RMSF of Complex_3, C- RMSF of Complex_6. The interpretation of the results is justified. Several significant fluctuations. The fluctuation primarily arises when the ligand interacts with the protein residues. Complex_1 exhibits three significant fluctuations, which signify the contact between the ligand molecule and the protein. Complex_3 and Complex_6 exhibit significant temporal fluctuations. The overall comparison reveals significant fluctuations, although they do not exceed 0.4 nm. (TIF) [file pone.0304490.s005.tif]

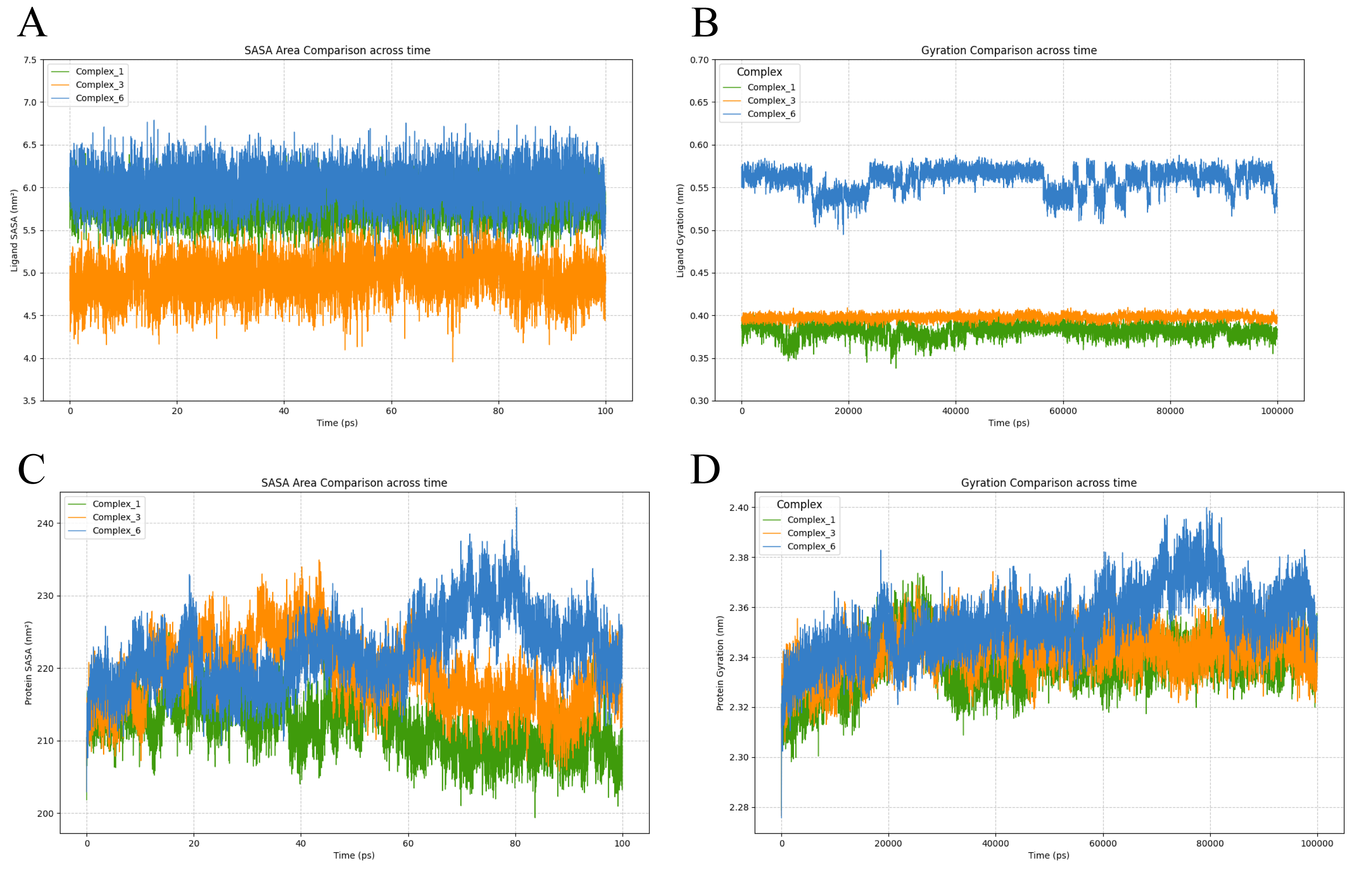

Supplement: S6 Fig — The green, orange, and blue colours represent Complex_1, Complex_3, and Complex_6, respectively. A) The SASA value of the ligand across 100 ns displayed lower solvent access. B) The gyration of the ligand results indicates that Complex_1 and Complex_3 are located within a range of 0.40 nm, while Complex_6 averages 0.55 nm. Additionally, Complex_6 showed irregularity in the radius of gyration, notably around 20 ns, indicating changes in atom dispersion around an axis. (TIF) [file pone.0304490.s006.tif]
