# Supplementary material for: A computational approach to identify phytochemicals as potential inhibitor of acetylcholinesterase: Molecular docking, ADME profiling and molecular dynamics simulations
Source: PLoS One. 2024 Jun 4;19(6):e0304490. doi: 10.1371/journal.pone.0304490 (PMC11149856; doi:10.1371/journal.pone.0304490)
Supplement: S1 Table — (DOCX) [file pone.0304490.s007.docx]

**S1 Table. The Active side residues.**

| Sequence ID | 70 | 71 | 73 | 82 | 85 | 86 | 87 | 119 | 120 | 121 | 123 | 124 |
| --- | --- | --- | --- | --- | --- | --- | --- | --- | --- | --- | --- | --- |
| Amino acid | GLN | TYR | ASP | THR | TRP | ASN | PRO | GLY | GLY | GLY | TYR | SER |
| Sequence ID | 125 | 129 | 132 | 201 | 202 | 235 | 285 | 288 | 292 | 293 | 294 | 295 |
| Amino acid | GLY | LEU | TYR | GLU | SER | TRP | TRP | LEU | SER | VAL | PHE | ARG |
| Sequence ID | 296 | 336 | 337 | 340 | 446 | 447 | 448 | 450 |  |  |  |  |
| Amino acid | PHE | TYR | PHE | TYR | HIS | GLY | TYR | ILE |  |  |  |  |
