# Supplementary material for: A computational approach to identify phytochemicals as potential inhibitor of acetylcholinesterase: Molecular docking, ADME profiling and molecular dynamics simulations
Source: PLoS One. 2024 Jun 4;19(6):e0304490. doi: 10.1371/journal.pone.0304490 (PMC11149856; doi:10.1371/journal.pone.0304490)
Supplement: S2 Table — (DOCX) [file pone.0304490.s008.docx]

**S2 Table. The sequence alignment of 3D predicted structures.**

| 3D predicted | EDAELLVTVRGGRLRGIRLKTPGGPVSAFLGIPFAEPPMGPRRFLPPEPKQPWSGVVDATTFQ |
| --- | --- |
| RCSB PDB | EDAELLVTVRGGRLRGIRLKTPGGPVSAFLGIPFAEPPMGPRRFLPPEPKQPWSGVVDATTFQ |
| 3D predicted | SVCYQYVDTLYPGFEGTEMWNPNRELSEDCLYLNVWTPYPRPTSPTPVLVWIYGGGFYSGASS |
| RCSB PDB | SVCYQYVDTLYPGFEGTEMWNPNRELSEDCLYLNVWTPYPRPTSPTPVLVWIYGGGFYSGASS |
| 3D predicted | LDVYDGRFLVQAERTVLVSMNYRVGAFGFLALPGSREAPGNVGLLDQRLALQWVQENVAAFGG |
| RCSB PDB | LDVYDGRFLVQAERTVLVSMNYRVGAFGFLALPGSREAPGNVGLLDQRLALQWVQENVAAFGG |
| 3D predicted | DPTSVTLFGESAGAASVGMHLLSPPSRGLFHRAVLQSGAPNGPWATVGMGEARRRATQLAHLV |
| RCSB PDB | DPTSVTLFGESAGAASVGMHLLSPPSRGLFHRAVLQSGAPNGPWATVGMGEARRRATQLAHLV |
| 3D predicted | GCPPGGTGGNDTELVACLRTRPAQVLVNHEWHVLPQESVFRFSFVPVVDGDFLSDTPEALINA |
| RCSB PDB | GCPP-----NDTELVACLRTRPAQVLVNHEWHVLPQESVFRFSFVPVVDGDFLSDTPEALINA |
| 3D predicted | GDFHGLQVLVGVVKDEGSYFLVYGAPGFSKDNESLISRAEFLAGVRVGVPQVSDLAAEAVVLH |
| RCSB PDB | GDFHGLQVLVGVVKDEGSYFLVYGAPGFSKDNESLISRAEFLAGVRVGVPQVSDLAAEAVVLH |
| 3D predicted | YTDWLHPEDPARLREALSDVVGDHNVVCPVAQLAGRLAAQGARVYAYVFEHRASTLSWPLWMG |
| RCSB PDB | YTDWLHPEDPARLREALSDVVGDHNVVCPVAQLAGRLAAQGARVYAYVFEHRASTLSWPLWMG |
| 3D predicted | VPHGYEIEFIFGIPLDPSRNYTAEEKIFAQRLMRYWANFARTGDPNEPRDPKAPQWPPYTAGA |
| RCSB PDB | VPHGYEIEFIFGIPLDPSRNYTAEEKIFAQRLMRYWANFARTGDPNEPRDAP--QWPPYTAGA |
| 3D predicted | QQYVSLDLRPLEVRRGLRAQACAFWNRFLPKLLSA |
| RCSB PDB | QQYVSLDLRPLEVRRGLRAQACAFWNRFLPKLLSA |
