# Supplementary material for: A computational approach to identify phytochemicals as potential inhibitor of acetylcholinesterase: Molecular docking, ADME profiling and molecular dynamics simulations
Source: PLoS One. 2024 Jun 4;19(6):e0304490. doi: 10.1371/journal.pone.0304490 (PMC11149856; doi:10.1371/journal.pone.0304490)
Supplement: S3 Table — (DOCX) [file pone.0304490.s009.docx]

**S3 Table. The geometrical analysis of 3D predicted protein.**

| Protein Geometry | Poor rotamers | 5 | 1.15% | Goal: <0.3% |
| --- | --- | --- | --- | --- |
|  | Favored rotamers | 418 | 96.09% | Goal: >98% |
|  | Ramachandran outliers | 0 | 0.00% | Goal: <0.05% |
|  | Ramachandran favored | 519 | 96.65% | Goal: >98% |
|  | Rama distribution Z-score | 0.45 ± 0.35 | | Goal: abs(Z score) < 2 |
|  | Cβ deviations >0.25Å | 0 | 0.00% | Goal: 0 |
|  | Bad bonds: | 1 / 4311 | 0.02% | Goal: 0% |
|  | Bad angles: | 28 / 5895 | 0.47% | Goal: <0.1% |
| Peptide Omegas | Cis Prolines: | 2 / 47 | 4.26% | Expected: ≤1 per chain, or ≤5% |
| Low-resolution Criteria | CaBLAM outliers | 8 | 1.5% | Goal: <1.0% |
|  | CA Geometry outliers | 2 | 0.37% | Goal: <0.5% |
| Additional validations | Chiral volume outliers | 0/626 | |  |
