# Supplementary material for: A computational approach to identify phytochemicals as potential inhibitor of acetylcholinesterase: Molecular docking, ADME profiling and molecular dynamics simulations
Source: PLoS One. 2024 Jun 4;19(6):e0304490. doi: 10.1371/journal.pone.0304490 (PMC11149856; doi:10.1371/journal.pone.0304490)
Supplement: S4 Table — (DOCX) [file pone.0304490.s010.docx]

**S4 Table. ADME analysis of screened chemical compounds.**

| Sl no | CID | IUPAC Name | Heavy atoms | Aromatic heavy atoms | Rotatable bonds | H-bond acceptors | H-bond donors | XLOGP3 | WLOGP | MLOGP | Synthetic Accessibility |  |
| --- | --- | --- | --- | --- | --- | --- | --- | --- | --- | --- | --- | --- |
| **Rivastigmine similar structures** | | | | | | | | | | | | |
| 1 | 77991 | Rivastigmine (control) | 18 | 6 | 6 | 3 | 0 | 2.29 | 2.44 | 2.34 | 2.73 |  |
| 2 | 70266158 | [2-[1-(azetidin-1-yl)ethyl]phenyl] N,N-dimethylcarbamate | 18 | 6 | 5 | 3 | 0 | 2.05 | 1.81 | 1.94 | 2.57 |  |
| 3 | 66717459 | [3-[(1S)-1-(dimethylamino)ethyl]-2-tritiophenyl] N-ethyl-N-methylcarbamate | 25 | 12 | 8 | 3 | 0 | 4.31 | 4.05 | 3.76 | 3.54 |  |
| 4 | 42604975 | [3-[(1S)-1-[methyl-[(1S)-1-phenylethyl]amino]ethyl]phenyl] N-ethyl-N-methylcarbamate | 25 | 12 | 8 | 3 | 0 | 4.18 | 4.24 | 3.76 | 3.48 |  |
| 5 | 129309692 | [3-[1-[[(1S)-1-cyclohexa-1,3-dien-1-ylethyl]-methylamino]ethyl]phenyl] N-ethyl-N-methylcarbamate | 25 | 6 | 8 | 3 | 0 | 3.98 | 4.47 | 3.45 | 4.23 |  |
| 6 | 68377091 | [3-[(1S)-1-(dimethylamino)ethyl]phenyl] N-ethynyl-N-[(2R)-1-phenylpropan-2-yl]carbamate | 26 | 12 | 8 | 3 | 0 | 4.69 | 4.09 | 3.9 | 3.89 |  |
| 7 | 144066490 | [3-[1-(dimethylamino)ethyl]phenyl] N-methyl-N-[(2R)-1-phenylpropan-2-yl]carbamate | 25 | 12 | 8 | 3 | 0 | 4.31 | 4.05 | 3.76 | 3.54 |  |
| 8 | 10989924 | [3-(1-methylpiperidin-2-yl)phenyl] N,N-diethylcarbamate | 21 | 6 | 6 | 3 | 0 | 3.13 | 2.98 | 2.69 | 2.98 |  |
| 9 | 11359764 | [3-[(1S)-1-[methyl(trideuterio(113C)methyl)amino]ethyl]phenyl] N-methyl-N-(1,1,2,2,2-pentadeuterio(213C)ethyl)carbamate | 25 | 12 | 8 | 3 | 0 | 4.31 | 4.05 | 3.76 | 3.54 |  |
| 10 | 46898202 | [3-(1-piperidin-1-ylethyl)phenyl] N,N-diethylcarbamate | 25 | 12 | 8 | 3 | 0 | 4.31 | 4.05 | 3.76 | 3.54 |  |
| 11 | 149047000 | [3-[1-(dimethylamino)cyclopropyl]phenyl] N-ethyl-N-methylcarbamate | 19 | 6 | 6 | 3 | 0 | 2.23 | 2.52 | 2.2 | 2.33 |  |
| 12 | 144474639 | [3-[(1S)-1-[[(1S)-1-cyclohexa-2,4-dien-1-ylethyl]-methylamino]ethyl]phenyl] N-ethyl-N-methylcarbamate | 25 | 6 | 8 | 3 | 0 | 4.46 | 4.33 | 3.45 | 4.47 |  |
| 13 | 21767521 | 7-[1-(dimethylamino)ethyl]-3-methyl-5,6-dihydro-4H-1,3-benzoxazocin-2-one | 19 | 6 | 2 | 3 | 0 | 2.31 | 1.98 | 2.2 | 3.03 |  |
| 14 | 21767510 | 6-[1-(dimethylamino)ethyl]-3-methyl-4,5-dihydro-1,3-benzoxazepin-2-one | 18 | 6 | 2 | 3 | 0 | 1.96 | 1.59 | 1.94 | 3.26 |  |
| 15 | 25204947 | [3-[(1S)-1-(dimethylamino)ethyl]phenyl] N-methyl-N-[(2S)-1-phenylpropan-2-yl]carbamate | 25 | 12 | 8 | 3 | 0 | 4.31 | 4.05 | 3.76 | 3.54 |  |
| 16 | 72816136 | [3-[1-(dimethylamino)ethyl]phenyl] N-methyl-N-(1-phenylpropan-2-yl)carbamate | 25 | 12 | 8 | 3 | 0 | 4.31 | 4.05 | 3.76 | 3.54 |  |
| 17 | 13955119 | [2-[1-(dimethylamino)ethyl]phenyl] N,N-dimethylcarbamate | 17 | 6 | 5 | 3 | 0 | 1.73 | 2.05 | 2.07 | 2.54 |  |
| 18 | 141557115 | [3-[1-(dimethylamino)pentyl]phenyl] acetate | 18 | 6 | 7 | 3 | 0 | 3.34 | 3.08 | 3.04 | 2.52 |  |
| 19 | 21767515 | 9-[1-(dimethylamino)ethyl]-3-methyl-5,6-dihydro-4H-1,3-benzoxazocin-2-one | 19 | 6 | 2 | 3 | 0 | 2.31 | 1.98 | 2.2 | 2.94 |  |
| 20 | 21767496 | 5-[1-(dimethylamino)ethyl]-3-methyl-4H-1,3-benzoxazin-2-one | 17 | 6 | 2 | 3 | 0 | 1.49 | 1.4 | 1.68 | 3.07 |  |
| 21 | 10935608 | [2-(1-piperidin-1-ylethyl)phenyl] N,N-diethylcarbamate | 22 | 6 | 7 | 3 | 0 | 3.5 | 3.37 | 2.93 | 3.03 |  |
| 22 | 10924256 | [3-(piperidin-1-ylmethyl)phenyl] N,N-diethylcarbamate | 21 | 6 | 7 | 3 | 0 | 3.63 | 2.98 | 2.69 | 2.41 |  |
| 23 | 144474633 | [3-[(2S)-1-(dimethylamino)propan-2-yl]phenyl] N-ethyl-N-methylcarbamate | 19 | 6 | 7 | 3 | 0 | 2.68 | 2.8 | 2.59 | 2.86 |  |
| 24 | 25230721 | [3-[(1S)-1,2,2,2-tetradeuterio-1-(dimethylamino)ethyl]phenyl] N-ethyl-N-methylcarbamate | 18 | 6 | 6 | 3 | 0 | 2.29 | 2.44 | 2.34 | 2.73 |  |
| 25 | 51037855 | [3-[(1S)-1,2,2,2-tetradeuterio-1-(dimethylamino)(213C)ethyl]phenyl] N-ethyl-N-methylcarbamate | 18 | 6 | 6 | 3 | 0 | 2.29 | 2.44 | 2.34 | 2.73 |  |
| 26 | 51038065 | [3-[(1S)-1-[methyl(trideuterio(113C)methyl)amino]ethyl]phenyl] N-ethyl-N-methylcarbamate | 18 | 6 | 6 | 3 | 0 | 2.29 | 2.44 | 2.34 | 2.73 |  |
| 27 | 21767507 | [3-[(1S)-1-[methyl(trideuterio(113C)methyl)amino]ethyl]phenyl] N-methyl-N-(1,1,2,2,2-pentadeuterio(213C)ethyl)carbamate | 18 | 6 | 2 | 3 | 0 | 1.96 | 1.59 | 1.94 | 3.21 |  |
| 28 | 9823072 | [3-[(1S)-1-(dimethylamino)ethyl]-2-tritiophenyl] N-ethyl-N-methylcarbamate | 19 | 6 | 7 | 3 | 0 | 2.66 | 2.83 | 2.59 | 2.83 |  |
| 29 | 53705187 | [2-[[ethyl(methyl)amino]methyl]phenyl] N,N-dimethylcarbamate | 17 | 6 | 6 | 3 | 0 | 1.7 | 2.05 | 2.07 | 2.06 |  |
| 30 | 97357026 | [3-[(1R)-1-(dimethylamino)ethyl]phenyl] N,N-diethylcarbamate | 19 | 6 | 7 | 3 | 0 | 2.66 | 2.83 | 2.59 | 2.83 |  |
| 31 | 11066683 | [3-(1-piperidin-1-ylethyl)phenyl] N,N-diethylcarbamate | 22 | 6 | 7 | 3 | 0 | 3.5 | 3.37 | 2.93 | 3.01 |  |
| 32 | 25230725 | [3-[(1S)-1-[bis(trideuteriomethyl)amino]-1,2,2,2-tetradeuterioethyl]-2,4,5,6-tetradeuteriophenyl] N-(1,1,2,2,2-pentadeuterioethyl)-N-(trideuteriomethyl)carbamate | 18 | 6 | 6 | 3 | 0 | 2.29 | 2.44 | 2.34 | 2.73 |  |
| 33 | 144198864 | (1S)-1-(3-methoxyphenyl)-N,N-dimethylpropan-1-amine | 14 | 6 | 4 | 2 | 0 | 2.66 | 2.38 | 2.4 | 1.94 |  |
| 34 | 67474850 | [3-[(1S)-1-(dimethylamino)ethyl]-4-fluorophenyl] N-ethyl-N-methylcarbamate | 19 | 6 | 6 | 4 | 0 | 2.39 | 2.99 | 2.73 | 2.94 |  |
| 35 | 10999871 | [3-(piperidin-1-ylmethyl)phenyl] N,N-dimethylcarbamate | 19 | 6 | 5 | 3 | 0 | 2.9 | 2.2 | 2.2 | 2.12 |  |
| 36 | 10586926 | [3-[(1S)-1-(dimethylamino)ethyl]-2-tritiophenyl] N-ethyl-N-methylcarbamate | 18 | 6 | 6 | 3 | 0 | 2.29 | 2.44 | 2.34 | 2.73 |  |
| 37 | 71316042 | [3-(1-piperidin-1-ylethyl)phenyl] N,N-diethylcarbamate | 19 | 6 | 7 | 3 | 0 | 2.66 | 2.83 | 2.59 | 2.83 |  |
| 38 | 745584 | [2-[(dimethylamino)methyl]phenyl] N,N-dimethylcarbamate | 16 | 6 | 5 | 3 | 0 | 1.33 | 1.66 | 1.8 | 1.94 |  |
| 39 | 25230720 | [2-deuterio-3-[(1S)-1-[dideuteriomethyl(methyl)amino]ethyl]phenyl] N-ethyl-N-methylcarbamate | 18 | 6 | 6 | 3 | 0 | 2.29 | 2.44 | 2.34 | 2.73 |  |
| 40 | 25230723 | [3-[(1S)-1-(dimethylamino)ethyl]phenyl] N-ethyl-N-(trideuteriomethyl)carbamate | 18 | 6 | 6 | 3 | 0 | 2.29 | 2.44 | 2.34 | 2.73 |  |
| 41 | 25230724 | [3-[(1S)-1-(dimethylamino)ethyl]phenyl] N-methyl-N-(1,1,2,2,2-pentadeuterioethyl)carbamate | 18 | 6 | 6 | 3 | 0 | 2.29 | 2.44 | 2.34 | 2.73 |  |
| 42 | 51037853 | [3-[(1S)-1,2,2,2-tetradeuterio-1-(dimethylamino)(113C)ethyl]phenyl] N-ethyl-N-methylcarbamate | 18 | 6 | 6 | 3 | 0 | 2.29 | 2.44 | 2.34 | 2.73 |  |
| 43 | 51038067 | [3-[(1S)-1-[methyl(trideuterio(113C)methyl)amino]ethyl]phenyl] N-methyl-N-(1,1,2,2,2-pentadeuterio(213C)ethyl)carbamate | 18 | 6 | 6 | 3 | 0 | 2.29 | 2.44 | 2.34 | 2.73 |  |
| 44 | 77991 | [3-(1-piperidin-1-ylethyl)phenyl] N,N-diethylcarbamate | 18 | 6 | 6 | 3 | 0 | 2.29 | 2.44 | 2.34 | 2.73 |  |
| 45 | 92044359 | [3-[(1R)-1-[bis(trideuteriomethyl)amino]ethyl]phenyl] N-ethyl-N-methylcarbamate | 0 | 0 | 0 | 3 | 0 | 2.29 | 2.44 | 2.34 | 2.73 |  |
| **Tacrine similar structures** | | | | | | | | | | | | |
| 46 | 1935 | Tacrine (control) | 15 | 10 | 0 | 1 | 1 | 2.71 | 2.7 | 3.4 | 2.08 |  |
| 47 | 18403988 | 2-naphthalen-2-ylquinolin-4-amine | 21 | 20 | 1 | 1 | 1 | 4.42 | 4.65 | 3.89 | 2.17 |  |
| 48 | 149800 | N-benzylacridin-9-amine | 22 | 20 | 3 | 1 | 1 | 4.9 | 4.66 | 3.35 | 1.65 |  |
| 49 | 402658 | 12-azatetracyclo[9.8.0.02,7.013,18]nonadeca-1(19),2,4,6,11,13,15,17-octaen-19-amine | 20 | 16 | 0 | 1 | 1 | 3.82 | 3.98 | 3.42 | 2.83 |  |
| 50 | 54474520 | 3-[2-(7-fluoroquinolin-2-yl)ethenyl]aniline | 20 | 16 | 2 | 2 | 1 | 3.91 | 4.34 | 3.58 | 2.45 |  |
| 51 | 3438772 | 2-phenyl-4-pyrrolidin-1-ylquinoline | 21 | 16 | 2 | 1 | 0 | 4.46 | 4.12 | 3.93 | 2.19 |  |
| 52 | 18934490 | N-phenylacridin-1-amine | 21 | 20 | 2 | 1 | 1 | 4.75 | 5.13 | 3.65 | 2.12 |  |
| 53 | 11492743 | 4-fluoro-2-(6-fluoro-4-methylquinolin-2-yl)aniline | 20 | 16 | 1 | 3 | 1 | 3.74 | 4.92 | 3.33 | 2.32 |  |
| 54 | 129829335 | 10-sulfidoacridin-10-ium | 15 | 14 | 0 | 0 | 0 | -0.98 | 2.59 | 3.26 | 1.29 |  |
| 55 | 164587579 | 2-benzyl-6-fluoroquinolin-4-amine | 19 | 16 | 2 | 2 | 1 | 3.57 | 3.98 | 3.23 | 2.15 |  |
| 56 | 130408026 | 2-(7-fluoro-2-phenylquinolin-3-yl)ethanamine | 20 | 16 | 3 | 3 | 1 | 3.27 | 3.96 | 3.04 | 2.37 |  |
| 57 | 22395290 | 2-[(E)-2-phenylethenyl]quinolin-4-amine | 19 | 16 | 2 | 1 | 1 | 3.81 | 3.78 | 3.04 | 2.31 |  |
| 58 | 69799851 | 2-(2-phenylethenyl)quinolin-4-amine | 19 | 16 | 2 | 1 | 1 | 3.81 | 3.78 | 4 | 2.31 |  |
| 59 | 696663 | 12-azatetracyclo[9.8.0.02,7.013,18]nonadeca-1(19),2,4,6,11,13,15,17-octaen-19-amine | 21 | 16 | 2 | 2 | 1 | 4.96 | 5.46 | 3.35 | 2.25 |  |
| 60 | 402666 | 19-azatetracyclo[9.8.0.02,7.013,18]nonadeca-1(19),2,4,6,11,13,15,17-octaen-12-amine | 20 | 16 | 0 | 1 | 1 | 3.98 | 3.98 | 3.4 | 2.79 |  |
| 61 | 10587156 | 6-fluoro-2-(2-fluorophenyl)quinolin-4-amine | 19 | 16 | 1 | 3 | 1 | 3.37 | 4.61 | 3.81 | 2.21 |  |
| 62 | 1504001 | 2-phenyl-4-piperidin-1-ylquinoline | 22 | 16 | 2 | 1 | 0 | 4.82 | 4.51 | 3.01 | 2.27 |  |
| 63 | 164587580 | 2-(2-fluorophenyl)quinolin-4-amine | 18 | 16 | 1 | 2 | 1 | 3.27 | 4.05 | 3.82 | 2.13 |  |
| 64 | 60598 | 9-(4-methylpiperidin-1-yl)-1,2,3,4-tetrahydroacridine | 21 | 10 | 1 | 1 | 0 | 4.8 | 3.97 | 2.62 | 2.62 |  |
| 65 | 4452632 | 3-quinolin-2-ylaniline | 17 | 16 | 1 | 1 | 1 | 3.22 | 3.49 | 2.7 | 1.95 |  |
| 66 | 7742109 | (NZ)-N-(1-phenyl-2-quinolin-2-ylethylidene)hydroxylamine | 20 | 16 | 3 | 3 | 1 | 3.76 | 3.66 | 2.66 | 2.49 |  |
| 67 | 12102730 | 2,4-dimethylbenzo[h]quinolin-10-amine | 17 | 14 | 0 | 1 | 1 | 3.52 | 3.6 | 2.67 | 1.61 |  |
| 68 | 21998 | 10-methylacridin-10-ium-9-amine | 16 | 14 | 0 | 0 | 1 | 2.98 | 2.41 | 3.01 | 1.39 |  |
| 69 | 45599224 | 12-azatetracyclo[9.8.0.02,7.013,18]nonadeca-1(19),2,4,6,11,13,15,17-octaen-19-amine | 18 | 16 | 1 | 2 | 1 | 3.27 | 4.05 | 3.4 | 2.04 |  |
| 70 | 45599463 | 5,7-difluoro-2-phenylquinolin-4-amine | 19 | 16 | 1 | 3 | 1 | 3.37 | 4.61 | 4 | 2.28 |  |
| 71 | 22334541 | N-(3-fluorophenyl)-2,3-dihydro-1H-cyclopenta[b]quinolin-9-amine | 21 | 16 | 2 | 2 | 1 | 4.52 | 5.03 | 3.5 | 2.54 |  |
| 72 | 11737199 | 2-(2-fluorophenyl)quinolin-4-amine | 20 | 16 | 2 | 2 | 0 | 4.08 | 4.53 | 2.87 | 2.26 |  |
| 2 | 55045454 | 6-methyl-2-phenylquinolin-4-amine | 18 | 16 | 1 | 1 | 1 | 3.54 | 3.8 | 2.67 | 2.08 |  |
| 73 | 31633 | 10-methylacridin-10-ium-3-amine | 16 | 14 | 0 | 0 | 1 | -1.9 | 2.41 | 3.4 | 1.49 |  |
| 74 | 45599470 | 7,8-difluoro-2-phenylquinolin-4-amine | 19 | 16 | 1 | 3 | 1 | 3.37 | 4.61 | 3.01 | 2.31 |  |
| 75 | 45599222 | 6-fluoro-2-phenylquinolin-4-amine | 18 | 16 | 1 | 2 | 1 | 3.27 | 4.05 | 2.8 | 2.06 |  |
| 76 | 21828278 | 2,6-diphenylpyridin-4-amine | 19 | 18 | 2 | 1 | 1 | 4.14 | 4.01 | 3.17 | 2.25 |  |
| 77 | 21639083 | 12-azatetracyclo[9.8.0.02,7.013,18]nonadeca-1(19),2,4,6,11,13,15,17-octaen-19-amine | 18 | 10 | 0 | 2 | 1 | 3.6 | 3.87 | 3.5 | 2.94 |  |
| 78 | 43419931 | N-[(4-fluorophenyl)methyl]-2-methylquinolin-4-amine | 20 | 16 | 3 | 2 | 1 | 4.42 | 4.37 | 3.04 | 1.87 |  |
| 79 | 129641425 | 2-(2-phenylethenyl)quinolin-3-amine | 19 | 16 | 2 | 1 | 1 | 3.81 | 3.78 | 2.34 | 2.22 |  |
| 80 | 12394207 | 2-phenyl-4-piperidin-1-ylquinoline | 16 | 14 | 1 | 2 | 2 | 2.71 | 3 | 3.39 | 1.89 |  |
| 81 | 10980245 | 2-(2-fluorophenyl)quinolin-4-amine | 24 | 10 | 5 | 2 | 1 | 4.61 | 3.83 | MLOGP | 2.96 |  |
| **Galantamine similar structures** | | | | | | | | | | | | |
| 82 | 91042094 | 9-methoxy-4-prop-2-enyl-11-oxa-4-azatetracyclo[8.6.1.01,12.06,17]heptadeca-6(17),7,9,15-tetraene | 22 | 6 | 3 | 3 | 0 | 3.59 | 2.9 | 2.98 | 4.44 |  |
| 83 | 20706288 | 4,14-dimethyl-11-oxa-4 azatetracyclo[8.7.1.01,12.06,18]octadeca-6(18),7,9,15-tetraen-9-ol | 21 | 6 | 0 | 3 | 1 | 3.25 | 2.68 | 2.83 | 4.42 |  |
| **Phytochemicals** | | | | | | | | | | | | |
| 84 | 2353 | Berberine | 25 | 16 | 2 | 4 | 0 | 3.62 | 3.1 | 2.19 | 3.14 |  |
| 85 | 5315472 | Bisdemethoxycurcumin | 23 | 12 | 6 | 4 | 2 | 3.26 | 3.13 | 2.13 | 2.59 |  |
| 86 | 6916252 | Huperzine B | 19 | 6 | 0 | 2 | 2 | 0.63 | 1.6 | 2.54 | 4.32 |  |
| 87 | 854026 | Huperzine A | 18 | 6 | 0 | 2 | 2 | 0.01 | 1.89 | 2.21 | 4.26 |  |
| 88 | 160512 | Ar-Turmerone | 16 | 6 | 4 | 1 | 0 | 3.98 | 4.02 | 3.68 | 2.4 |  |
| 89 | 1253 | (-)-Selagine | 18 | 6 | 0 | 2 | 2 | 0.01 | 1.89 | 2.21 | 4.26 |  |
