# Supplementary material for: A computational approach to identify phytochemicals as potential inhibitor of acetylcholinesterase: Molecular docking, ADME profiling and molecular dynamics simulations
Source: PLoS One. 2024 Jun 4;19(6):e0304490. doi: 10.1371/journal.pone.0304490 (PMC11149856; doi:10.1371/journal.pone.0304490)
Supplement: S5 Table — (DOCX) [file pone.0304490.s011.docx]

**S5 Table. Molecular docking Study of primary screening by pyrx.**

| Ligand CID | Binding Affinity |
| --- | --- |
| **Rivistigmaine (Control)** | **-6.5** |
| 10978630, 11121243, 118266871, 122201388, 123974141, 58575674, 132520303, 141337671, 143733569, 154047697, 25230720, 57957504, 46844523, 51037853, 51038067, 52172101, 54347640, 92044359, 73357705, 745584, 77991, 807291, 89029043, 89185415, 124887483, 124887488, 25230723, 25230724 | -6.6 |
| 10586926, 10999871, 110293312, 121566300, 122684106, 129309606, 138714728, 141136311, 144198864, 144275922, 144489593, 146328534, 54525847, 67474850, 67612431, 71307612, 71316042, 77298751, 82023505, 82124462, 97103070 | -6.7 |
| 101486795, 119038415, 12105232, 143747662, 144066487, 150739543, 23037471, 23037475, 23645310, 25098268, 25230725, 47227, 59825404, 70301425, 82124167 | -6.8 |
| 100763010, 11066683, 11298747, 81373890, 141151203, 17329762, 21767524, 54348030, 56971803, 57701214, 57957289, 57957453, 58575697, 66685339, 71307596, 745596 | -6.9 |
| 102313934, 121005381, 134960463, 15547585, 20666915, 20791805, 42604976, 53705187, 57052307, 57145096, 57246079, 58575718, 68719143, 69484341, 97357026 | -7 |
| 100763006, 101437314, 103729548, 11277406, 58575733, 60717816, 66645870, 118266861, 141136309, 141136312, 144474633, 67474849, 81372879, 86334041, 21767507, 22025986, 25204941, 25205011, 97103071, 97357025, 9823072, 25230721, 25271873, 51037855, 51038065, 25230721, 25271873, 51037855, 51038065 | -7.1 |
| 9823072, 101779617, 101957245, 102437812, 10924256, 155749556, 162641534, 21767496, 21767515, 23037472, 52172100, 56930479, 56955216, 57036817, 58575693, 60630660, 74428300, 58575745, 10935608, 118266860 | -7.2 |
| 118266846, 124202028, 13955119, 141557115, 143733610, 23037473, 25095376, 95711614 | -7.3 |
| 103729543, 10947236, 21767500, 21767510, 23361258, 23361301, 46111, 50899395, 66575503, 86712426, 77146706, 2572816136, 204947 | -7.4 |
| 11110901, 11324419, 21767521, 46572, 60709205, 64991222, 68548559, 72801698 | -7.5 |
| 10860655, 10989924, 11359764, 122610642, 144474639, 149047000, 46780777, 46898202, 25204876 | -7.6 |
| 11337495, 144066490, 15189985, 60681147, 23037476, 25175200, 25204945, 68377091, 29973693, 58575642, 60655054, 72806893 | -7.7 |
| 11809309, 15189984, 15222798, 64593253, 95711613, 60681283 | -7.8 |
| 129309692, 56887809, 5744837 | -7.9 |
| 11404631, 25052431, 29973689, 42604975, 54201944 | -8 |
| 10062018, 11198180, 137320135, 44189032, 46780776, 52940766, 66717459, 70266158 | -8.1 |
| 103729569, 154400938, 50899285, 74817986 | -8.3 |
| 10882164, 127542734, 23037469, 89107701 | -8.4 |
| 61067661 | -8.5 |
| 102008667, 10664200 | -8.8 |
| 21857217, 72834171 | -9.0 |
| 11163193 | -9.1 |
| **Tacrine** | **-9.0** |
| 101166035, 10774819, 10980245, 11275885, 11384, 11622684, 12227971, 12394207, 129641425, 129829335, 21639083, 21828278, 21839643, 22600895, 23519, 242968, 31626, 129845974, 13433276, 14536417, 14983509, 153727925, 153963301, 15655497, 15818822, 18439823, 667620, 70841547, 828189, 86141464, 19081, 31627, 31633, 43419931, 45599222, 45599470, 54131910, 58046081, 147610 | -9.1 |
| 45599224, 45599463, 5297706, 55045454, 609927, 613020, 86670055, 11737199, 163228935, 163756450, 22334541, 2302710, 31624, 164587580, 18439896, 20919, 21998, 44339352, 31628, 11886, 130408026, 140124190, 14068992, 153676, 155827 | -9.2 |
| 101559789, 12102730, 87586132, 153529380, 153725961, 164587579, 12514010, 137191, 13816262, 141225376, 144834041, 14536415, 21298179, 21839636, 25135554, 38498, 42805, 612876, 7742109, 4452632, 45599465, 46661, 477874, 59246301, 60598, 85827101 | -9.3 |
| 151108050, 19031627, 4187112, 58798393, 612824, 102444189, 10587156, 11962076, 12820772, 42803, 44339281, 5758227, 71379433, 14536410, 148844, 149544, 1504001 | -9.4 |
| 402666, 4108827, 44339176, 44339289, 67253181, 696663, 10539341, 11021600, 11108968, 11913, 130408010, 14906154, 15706043, 163867686, 164679083, 19816709, 13120185, 134152700, 22395290, 23126521, 69799851 | -9.5 |
| 102449710, 10263956, 15365886, 45113380, 11492743, 13432122, 53347, 10493568 | -9.6 |
| 10162, 10243847, 13571817, 13986907, 21839649, 26608878, 30331917, 141328940, 18934490, 402664, 402664, 44338975, 44338975 | -9.7 |
| 10063333, 102501348, 15009586, 22637080, 3836807, 57116756, 154709770, 89552193, 12669577, 54474520 | -9.8 |
| 11715887, 146692, 149800, 21639122, 402658, 44537034, 16443313, 1933, 155824, 45599229, 54157621 | -9.9 |
| 11139460, 141397502, 5763302, 6144426, 21839654, 153354659 | -10.0 |
| 153354658, 6223897, 4878859, 402660 | -10.1 |
| 145982657, 71527480, 4854660 | -10.2 |
| 18403988 | -10.3 |
| 22231, 38499 | -10.4 |
| 66660021 | -10.5 |
| 161950989 | -11.1 |
| 147746724 | -11.2 |
| **Galandamine (Control)** | **-8.3** |
| 11869393, 12312560, 12313118, 130439232, 134990432, 144127672, 147876457, 67523087, 68012733, 58645927, 58645974, 58783295, 58783342, 58783373, 58783416, 58783462, 58872835, 59664531, 69415825, 69443053, 69446159, 70301174, 71129349, 71131725, 71209912, 71210897, 72422373, 72422393, 87613923, 87613935, 90714386, 90899470, 91204883, 59686480, 14803829, 154699493, 155562261, 20706288, 58235464, 71215714, 71220737, 71220767, 71220867 | -8.4 |
| 101030895, 10221208, 12312559, 130438429, 140453657, 146028054, 146030413, 147158172, 159131090, 159425850, 163093251, 906210, 163189375, 20706273, 443739, 49849704, 58645884, 90968554, 58645965, 58783231, 58783457, 59973727, 60039184, 91151722, 69168405, 69415428, 69416378, 69440353, 71210901 | -8.5 |
| 10084442, 11282165, 134990189, 138563267, 140467761, 143631539, 143631576, 146028053, 146028062, 58783335, 58783408, 58872820, 58966785, 59686474, 59686476, 68012764, 68892401, 69414827, 69417112, 71195094, 71209885, 73056462, 73297380, 73297384, 73303191, 87824349, 90959681, 91042094, 91524860, 91616923 | -8.6 |
| 11380243, 141884120, 146028082, 58783418, 58872800, 58872826, 59686477, 69414244, 69415855, 73297379, 75219936, 87614327, 89049518 | -8.7 |
| 101639268, 143631492, 162965348, 21076630, 402597, 58783241, 67523089, 69415013, 69415824, 69415963, 69500685, 71210855, 71210877, 71210890, 71215745, 87614078, 89140687 | -8.8 |
| 123582332, 14493587, 146030829, 58783469, 68891397, 69417538, 73847088, 87613924, 91351939, 58600505, 58645915, 58783256, 70214816, 71209907 | -8.9 |
| 140465339, 143631561, 14413746, 146028055, 58645889, 58783347, 68893326, 68894502, 71209906, 71220870, 72381541, 87613993, 87614303, 87614591, 89140692, 90734181, 91350415 | -9 |
| 57904026, 59294313, 69441695, 69452134, 71209881, 71210902, 87614082, 89049517, 89049519 | -9.1 |
| 57903999, 58872827, 71129553, 71210885, 71210893, 71220725, 87613901, 89298051 | -9.2 |
| 146028085, 146028090, 58645901, 58783414, 58783320 | -9.3 |
| 123857979, 87825198, 69419206 | -9.4 |
| 21076629, 69417137, 58783354, 89049533 | -9.5 |
| 71215773, 89232023 | -9.7 |
| 58645979 | -9.8 |
| 58783442 | -9.9 |
| 158979188, 58783376, 71210856 | -10.1 |
| 73303256 | -10.2 |
| **Donepezil (Control)** | **-9.9** |
| 89059282, 89059355, 89059318 | -10.4 |
| 89059338 | -10.3 |
| 89059357, 91262225 | -10.2 |
| 89059296, 89059324 | -9.9 |
| **Pythochemicals CID** |  |
| 2353 | -9.3 |
| 5315472 | -9.3 |
| 1742129 | -8.2 |
| 101115386 | -8.2 |
| 6916252 | -8.1 |
| 854026 | -7.9 |
| 160512 | -7.6 |
| 1253 | -6.9 |
| 10364 | -6.9 |
| 7462 | -6.8 |
| 17100 | -6.8 |
| 6989 | -6.7 |
