# Supplementary material for: A computational approach to identify phytochemicals as potential inhibitor of acetylcholinesterase: Molecular docking, ADME profiling and molecular dynamics simulations
Source: PLoS One. 2024 Jun 4;19(6):e0304490. doi: 10.1371/journal.pone.0304490 (PMC11149856; doi:10.1371/journal.pone.0304490)
Supplement: Attachment — Submitted filename: Reviewed_PONE-D-24-10565.pdf [file pone.0304490.s013.pdf]

PLOS ONE

A Computational Approach to Identify Phytochemicals as an inhibitor of Acetylcholinesterase: Molecular Docking, ADME profiling and Molecular Dynamics Simulations

--Manuscript Draft--

**Manuscript Number:**	PONE-D-24-10565
**Article Type:**	Research Article
**Full Title:**	A Computational Approach to Identify Phytochemicals as an inhibitor of Acetylcholinesterase: Molecular Docking, ADME profiling and Molecular Dynamics Simulations
**Short Title:**	Identification of Phytochemicals as an inhibitor of Acetylcholinesterase
**Corresponding Author:**	Ajit Ghosh<br>Shahjalal University of Science and Technology<br>Sylhet, Sylhet BANGLADESH
**Keywords:**	Alzheimer's disease, Acetylcholinesterase, Phytochemicals, Molecular docking, Molecular dynamic simulation.
**Abstract:**	Inhibition of acetylcholinesterase (AChE) is a crucial target in the treatment of Alzheimer’s disease (AD). Common anti-acetylcholinesterase drugs such as galantamine, rivastigmine, donepezil, and tacrine have significant inhibition potential. Due to side effects and safety concerns, we aimed to investigate a wide range of phytochemicals and structural analogues of these compounds. Compounds similar to established drugs, and phytochemicals were investigated as potential inhibitors for AChE in treating AD. A total of 2,270 compound libraries were generated for further analysis. Initial virtual screening was performed using Pyrx software, resulting in 638 molecules showing higher binding affinities compared to positive controls Tacrine (-9.0 kcal/mol), Donepezil (-7.3 kcal/mol), Galantamine (-8.3 kcal/mol), and Rivastigmine (-6.4 kcal/mol). Subsequently, ADME properties were assessed, including blood-brain barrier permeability and Lipinski’s rule of five violations, leading to 88 compounds passing the ADME analysis. Among the rivastigmine analogous, [3-(1-methylpiperidin-2-yl)phenyl] N,N-diethylcarbamate showed interaction with Tyr123, Tyr336, Tyr340, Phe337, Trp285 residues of AChE. Tacrine similar compounds, such as 4-amino-2-styrylquinoline, exhibited bindings with Tyr123, Phe337, Tyr336, Trp285, Trp85, Gly119, and Gly120 residues. A phytocompound bisdemethoxycurcumin showed interaction with Trp285, Tyr340, Trp85, Tyr71, and His446 residues of AChE with favourable binding. These findings underscore the potential of these compounds as novel inhibitors of AChE, offering insights into alternative therapeutic avenues for Alzheimer’s disease. Further investigation, including in vitro and in vivo studies, is needed to validate the efficacy, safety profiles, and therapeutic potential of these compounds for Alzheimer’s disease treatment.
**Order of Authors:**	Mahir Azmal, BSc
	Md. Sahadot Hossen, MSc
	Naimul Haque Shohan, BSc
	Md Rasid Taqui, MSc
	Abbeha Malik, MSc
	Ajit Ghosh, PhD
**Additional Information:**	
**Question**	**Response**
**Financial Disclosure**<br><br>Enter a financial disclosure statement that describes the sources of funding for the	The author(s) received no specific funding for this work.

Powered by Editorial Manager® and ProduXion Manager® from Aries Systems Corporation

work included in this submission. Review the submission guidelines for detailed requirements. View published research articles from *PLOS ONE* for specific examples.<br><br>This statement is required for submission and **will appear in the published article** if the submission is accepted. Please make sure it is accurate.<br><br>**Funded studies**<br>Enter a statement with the following details:<br>• Initials of the authors who received each award<br>• Grant numbers awarded to each author<br>• The full name of each funder<br>• URL of each funder website<br>• Did the sponsors or funders play any role in the study design, data collection and analysis, decision to publish, or preparation of the manuscript?<br><br>Did you receive funding for this work?	
**Competing Interests**<br><br>Use the instructions below to enter a competing interest statement for this submission. On behalf of all authors, disclose any competing interests that could be perceived to bias this work—acknowledging all financial support and any other relevant financial or non-financial competing interests.<br><br>This statement is required for submission and **will appear in the published article** if the submission is accepted. Please make sure it is accurate and that any funding sources listed in your Funding Information later in the submission form are also declared in your Financial Disclosure statement.<br><br>View published research articles from *PLOS ONE* for specific examples.	The authors have declared that no competing interests exist.

**NO authors have competing interests**<br><br>Enter: *The authors have declared that no competing interests exist.*<br><br>**Authors with competing interests**<br><br>Enter competing interest details beginning with this statement:<br><br>*I have read the journal's policy and the authors of this manuscript have the following competing interests: [insert competing interests here]*<br><br>* typeset	
**Ethics Statement**<br><br>Enter an ethics statement for this submission. This statement is required if the study involved:<br><br>• Human participants<br>• Human specimens or tissue<br>• Vertebrate animals or cephalopods<br>• Vertebrate embryos or tissues<br>• Field research<br><br>Write "N/A" if the submission does not require an ethics statement.<br><br>General guidance is provided below. Consult the submission guidelines for detailed instructions. **Make sure that all information entered here is included in the Methods section of the manuscript.**	N/A

**Format for specific study types**<br><br>**Human Subject Research (involving human participants and/or tissue)**<br>• Give the name of the institutional review board or ethics committee that approved the study<br>• Include the approval number and/or a statement indicating approval of this research<br>• Indicate the form of consent obtained (written/oral) or the reason that consent was not obtained (e.g. the data were analyzed anonymously)<br><br>**Animal Research (involving vertebrate animals, embryos or tissues)**<br>• Provide the name of the Institutional Animal Care and Use Committee (IACUC) or other relevant ethics board that reviewed the study protocol, and indicate whether they approved this research or granted a formal waiver of ethical approval<br>• Include an approval number if one was obtained<br>• If the study involved *non-human primates*, add *additional details* about animal welfare and steps taken to ameliorate suffering<br>• If anesthesia, euthanasia, or any kind of animal sacrifice is part of the study, include briefly which substances and/or methods were applied<br><br>**Field Research**<br><br>Include the following details if this study involves the collection of plant, animal, or other materials from a natural setting:<br>• Field permit number<br>• Name of the institution or relevant body that granted permission	
**Data Availability**<br><br>Authors are required to make all data underlying the findings described fully available, without restriction, and from the time of publication. PLOS allows rare exceptions to address legal and ethical concerns. See the PLOS Data Policy and FAQ for detailed information.	Yes - all data are fully available without restriction

A Data Availability Statement describing where the data can be found is required at submission. Your answers to this question constitute the Data Availability Statement and **will be published in the article**, if accepted.<br><br>**Important:** Stating 'data available on request from the author' is not sufficient. If your data are only available upon request, select 'No' for the first question and explain your exceptional situation in the text box.<br><br>Do the authors confirm that all data underlying the findings described in their manuscript are fully available without restriction?	
**Describe where the data may be found in full sentences. If you are copying our sample text, replace any instances of XXX with the appropriate details.**<br><br>• If the data are **held or will be held in a public repository**, include URLs, accession numbers or DOIs. If this information will only be available after acceptance, indicate this by ticking the box below. For example: *All XXX files are available from the XXX database (accession number(s) XXX, XXX.).*<br>• If the data are all contained **within the manuscript and/or Supporting Information files**, enter the following: *All relevant data are within the manuscript and its Supporting Information files.*<br>• If neither of these applies but you are able to provide **details of access elsewhere**, with or without limitations, please do so. For example:<br><br>*Data cannot be shared publicly because of [XXX]. Data are available from the XXX Institutional Data Access / Ethics Committee (contact via XXX) for researchers who meet the criteria for access to confidential data.*<br><br>*The data underlying the results presented in the study are available from (include the name of the third party*	All relevant data are within the manuscript and its Supporting Information files.

and contact information or URL).<br>• This text is appropriate if the data are owned by a third party and authors do not have permission to share the data.<br><br>* typeset	
Additional data availability information:	

Powered by Editorial Manager® and ProduXion Manager® from Aries Systems Corporation

Manuscript

Click here to access/download;Manuscript;AcHEi_manuscript.docx

A Computational Approach to Identify Phytochemicals as an inhibitor of Acetylcholinesterase: Molecular Docking, ADME profiling and Molecular Dynamics Simulations

Mahir Azmal[1], Md. Sahadot Hossen[1], Naimul Haque Shohan[1], Md Rasid Taqui[1], Abbeha Malik[2,*], Ajit Ghosh[1,*]

[1]Department of Biochemistry and Molecular Biology, Shahjalal University of Science and Technology, Sylhet 3114, Bangladesh, and

[2]Department of Bioinformatics, Institute of Biochemistry, Biotechnology and Bioinformatics, The Islamia University of Bahawalpur, Pakistan.

*Corresponding Author: abbeha.malik@iub.edu.pk, aghosh-bmb@sust.edu

Abstract

Inhibition of acetylcholinesterase (AChE) is a crucial target in the treatment of Alzheimer's disease (AD). Common anti-acetylcholinesterase drugs such as galantamine, rivastigmine, donepezil, and tacrine have significant inhibition potential. Due to side effects and safety concerns, we aimed to investigate a wide range of phytochemicals and structural analogues of these compounds. Compounds similar to established drugs, and phytochemicals were investigated as potential inhibitors for AChE in treating AD. A total of 2,270 compound libraries were generated for further analysis. Initial virtual screening was performed using Pyrx software, resulting in 638 molecules showing higher binding affinities compared to positive controls Tacrine (-9.0 kcal/mol), Donepezil (-7.3 kcal/mol), Galantamine (-8.3 kcal/mol), and Rivastigmine (-6.4 kcal/mol). Subsequently, ADME properties were assessed, including blood-brain barrier permeability and Lipinski's rule of five violations, leading to 88 compounds passing the ADME analysis. Among the rivastigmine analogous, [3-(1-methylpiperidin-2-yl)phenyl] N,N-diethylcarbamate showed interaction with Tyr123, Tyr336, Tyr340, Phe337, Trp285 residues of AChE. Tacrine similar compounds, such as 4-amino-2-styrylquinoline, exhibited bindings with Tyr123, Phe337, Tyr336, Trp285, Trp85, Gly119, and Gly120 residues. A phytocompound bisdemethoxycurcumin showed interaction with Trp285, Tyr340, Trp85, Tyr71, and His446 residues of AChE with favourable binding. These findings underscore the potential of these compounds as novel inhibitors of AChE, offering insights into alternative therapeutic avenues for Alzheimer's disease. Further investigation, including in vitro and in vivo studies, is needed to validate the efficacy, safety profiles, and therapeutic potential of these compounds for Alzheimer's disease treatment.

Keywords: Alzheimer's disease, Acetylcholinesterase, Phytochemicals, Molecular docking, Molecular dynamic simulation.

Introduction

Alzheimer's disease (AD) is a neurological disorder that leads to the deterioration of brain cells. It is the primary cause of dementia, a condition marked by a decline in cognitive abilities and a loss of independence in daily tasks (Breijyeh & Karaman, 2020). AD is characterized by a decline in the cholinergic system, resulting in reduced levels of acetylcholine in brain regions responsible for learning, memory, behaviour, and emotional responses (Anand et al., 2012). AD is neuropathologically defined by the presence of beta-amyloid (Aβ) plaques, neurofibrillary tangles, and degeneration or atrophy of the basal forebrain cholinergic neurons (Roberson & Harrell, 1997). Acetylcholinesterase (AChE), an enzyme that belongs to the serine hydrolase family, plays a vital role in breaking down acetylcholine (ACh) into choline and acetate. Therefore, maintaining normal cholinergic neurotransmission. In AD patients ACh degradation is amplified by the AChE in early stages. The use of enzymatic inhibition to reduce AChE activity has shown promise as a treatment strategy for AD (Du et al., 2018). The FDA-approved AChE enzyme inhibitors donepezil and rivastigmine are utilized for the treatment of mild to moderate AD. Tacrine was one of the AChE inhibitory drugs which had been banned since 2013. Both medications have adverse effects such as nausea, diarrhoea, loss of appetite, fainting, abdominal pain, and vomiting (Tayeb et al., 2012). Administration of tacrine (THA) for AD treatment leads to reversible hepatotoxicity in 30-50% of patients, as evidenced by an elevation in transaminase levels (Lagadic-Gossmann et al., 1998). Therefore, scientists are searching for more effective agents with fewer side effects (Scheltens et al., 2021).

Researchers have investigated natural resources for anti-AChE agents because they are safer than synthetic chemicals (Kim et al., 2010). Galantamine, a natural drug from *Galanthus woronowii*, is used to treat AD alongside other chemical drugs (Bartolucci et al., 2001). However, none of these medications have proven to be entirely effective in halting the advancement or formation of AD. To ameliorate the potential side effects and optimize the therapeutic efficacy of enzyme inhibition, compounds possessing structural similarities to FDA-approved drugs emerge as promising candidates (Birks & Harvey, 2003; Olin & Schneider, 2002; Onor et al., 2007). Ongoing research is being conducted to discover novel compounds derived from natural sources or FDA-approved drug-like compounds with anti-AChE properties (Pilger et al., 2001). Natural products derived from different plants are increasingly being recognized globally for their potential as AChE inhibitors (AChEi), making them a promising therapeutic option for the treatment of AD (Taqui et

al., 2022). Extensive research has identified a comprehensive list of plant-derived substances that inhibit AChE. The research on AChE inhibition-based treatment of AD has focused on this diverse range of phytochemicals due to the absence of promising, effective, and safe inhibitors (Kim et al., 2010; Sarkar et al., 2021).

Studies have demonstrated that memory-enhancing herbs such as *Enhydra fluctuans*, *Vanda roxburghii*, *Bacopa monnieri*, *Centella asiatica*, *Convolvulus pluricaulis*, and *Aegle marmelos* have acetylcholinesterase inhibitory and antioxidant properties. Acetylcholinesterase, the main cholinesterase in the brain that breaks down acetylcholine, shows greater specificity for acetylcholine. The findings indicate possible advantages for treating Alzheimer's disease (Lopa et al., 2021). This study aims to elucidate how human AChE is inhibited by the current FDA-approved drugs similar to structure analogues, as well as phytochemicals. Our study aimed to assess the *in-silico* assay results through docking, ADME simulation (RMSD, RMSF, Ligand properties), PCA, and DCCM, comparing them with FDA-approved drugs (donepezil, galantamine, rivastigmine), a selective AChE inhibitor employed in current AD therapy.

Materials and Methods

Ligand Selection

Ligand library 1: Similar structure selection

The rationale behind constructing library 1 (Similar structure search) was two-sided. Firstly, compounds with analogous structures might be able to show a similar kind of effect to some extent. Secondly, studies have reported mild to severe adverse effects upon their administration and among them. Each of the four compounds was used as a query in the PubChem database followed by a similar structure search.

Ligand library 2: Dr. Duke database search for phytochemicals

Phytochemicals, known for their anti-AChE and anti- Butyrylcholinesterase (BChE) activities, were identified through a literature review of medicinal plants. Scientific names were queried in Dr. Duke's Phytochemical and Ethnobotanical Databases (https://phytochem.nal.usda.gov/). Compound names were then searched in PubChem for 3-D structure retrieval.

Selection of target protein and protein preparation

The RSCB-PDB database (https://www.rcsb.org) was utilized to search for the target protein, human acetylcholinesterase protein (PDB ID: 4M0E) with a lower X-ray resolution (2.00 Å). Several gaps were spotted while checking the structure with PyMol. Both the docking and simulation processes were vulnerable to interference from missing residues. To avoid any subsequent anomaly in docking and molecular dynamics simulation the spotted missing residues were repaired. To ensure the missing residues I-tasser (https://zhanggroup.org/I-TASSER/) a web-based server was used to predict the 3D structure of protein. The FASTA sequence was retrieved from the RCSB PDB database and used to build the predicted structure. The geometry analysis was performed using the MolProbity server (http://molprobity.biochem.duke.edu/), and the overall geometry and Ramachandran plots were analyzed.

Active site prediction

The active region on the surface of the protein that performs protein function is known as a protein-ligand binding site. To avoid blind docking the specific amino acid residues (Table S1) of protein-ligand interaction were predicted using CASTP v3.0 (http://sts.bioe.uic.edu/castp/calculation.html).

Molecular docking of primarily selected molecules.

PyRx 0.8 was used for the initial virtual screening (Dallakyan & Olson, 2015a). The protein was retrieved from the I-tasser website in PDB format after homology modelling and ligands were downloaded from the PubChem of NCBI (https://pubchem.ncbi.nlm.nih.gov) one by one in SDF file format.

The target protein was loaded in Pyrx 0.8 and converted into macromolecules. The similar structures of tacrine, donepezil, rivastigmine and galantamine (considered as controls) along with phytochemicals were loaded in the PyRx virtual screening tool. After energy minimization, it was converted into a pdbqt file. All the parameters and grid box positioned at some standard value (Centre box: X = -0.9600, Y = -38.1677, Z = 34.2085) and the dimensions in Angstrom were X = 58.7652, Y = 60.0782 and Z = 65.867. Later, the docking results were screened for binding affinity and then all the generated possible docked conformations were stored in CSV format (Dallakyan & Olson, 2015b). Only those conformations that interacted specifically with the active-site

residues of the target protein targeted protein were selected and further detailed interactions were explored through Discovery Studio and PyMOL.

ADME Profiling

The SwissADME (http://www.swissadme.ch/index.php) server was utilized to conduct ADME profiling. Canonical smiles of ligands were required for conducting ADME analysis. To perform ADME profiling, the canonical smiles of all the ligands were uploaded as input on the SwissADME server. The entirety of the data was acquired in the CSV (comma-separated value) format. The subsequent sorting procedure was conducted according to the permeability of the blood-brain barrier, greater binding affinity, violations of drug-likeness violation (Lipinski, Ghose, Veber, Egan, Muggue), and oral bioactivity (lipophilicity, flexibility, solubility, instability, size) (Daina et al., 2017).

Molecular Re-docking performance

Re-docking was performed by the AutoDock Vina tool for the reliability of the software, and consistency of the docking algorithm. The target protein was converted into pdbqt. The parameters and grid box were positioned at some standard value (Centre box: X =106.848, Y = 43.703, Z = 18.797) and the dimensions of Box in Angstrom were X = 126, Y = 116 and Z = 122. Subsequently, the docking results were screened for binding affinity and generated all possible docked conformations were stored in the pdbqt file. Docking results were reported as a negative score in kcal/mol where the lowest docking score indicates the highest binding affinity (Kuntz, 1992).

Molecular Dynamic Simulation

Protein-ligand interaction stability during macromolecule structure-to-function transitions was studied using molecular dynamics. The Desmond software, developed by Schrödinger LLC, enabled the execution of molecular dynamics (MD) simulations that lasted for a duration of 100 nanoseconds. The simulations, utilizing Newton's classical equation of motion, monitored the path of atoms as they moved through time. The receptor-ligand complex was subjected to preprocessing using Maestro's Protein Preparation Wizard, which included optimization and minimization procedures. The system was prepared using the System Builder tool, employing the Transferable

Intermolecular Interaction Potential 3 Points (TIP3P) solvent model within an orthorhombic box. The simulation was governed by the OPLS 2005 force field, and counter ions were introduced to maintain model neutrality. A 0.15 M sodium chloride (NaCl) solution was added to replicate the conditions found in the body. The simulations were conducted using the Number of particles (N), Pressure (P), and Temperature (NPT) ensemble, with a temperature of 300 K and a pressure of 1 atm. Before the simulation, the models underwent a process of relaxation. The trajectories were recorded at intervals of 100 picoseconds. The stability was evaluated by comparing the root mean square deviation (RMSD), root mean square fluctuation (RMSF), Ligand properties (radius of Gyration, Molecular surface area, hydrogen bond etc.), PCA and DCCM of the protein and ligand during the entire simulation (Malik et al., 2023; Rathod et al., 2023).

Results

Ligand library construction

The number of similar structure compounds were massive; however, considering the facts about drug-likeness several criteria were optimized to select the best suited structures. A total of 2252 similar compounds (library 1) and 18 phytochemicals (library 2) were primarily selected for virtual screening based on the selection criteria (Table 1).

Table 1: Primary selection Criteria for similar structure compounds

Compound name/Criteria	Molecular Weight G/MOL [Min-Max]	Rotatable Bond Count [Min-Max]	Heavy Atom Count [Min-Max]	H-Bond Donor Count [Min-Max]	H-Bond Acceptor Count [Min-Max]	Polar Area, [Angstrom sq] [Min-Max]	Complexity [Min-Max]	XLOGP [Min-Max]
Tacrine	147-467	0-9	12-30	0-4	0-10	4.9-104	144-494	1-5
Donepezil	289-479	4-9	21-35	0-2	2-8	26.3-119	366-776	2-5
Rivastigmine	179.26-479	2-9	13-30	0-3	2-7	12.2-112	147-497	-0.3-4.7
Galantamine	245-445	0-9	18-32	0-4	2-9	18.5-128	326-766	-2.4-4

3D structure prediction

The I-tasser gave a modelled structure which is like the 4M0E pdb (Fig. 1). The alignment of the sequence of amino acids is provided to verify the residues, with further sequence alignment and geometry details (Table S2 and Table S3). The Ramachandran plot (Fig. S1) shows the statistical distribution of the combinations of the backbone dihedral angles ϕ and ψ. In theory, the allowed regions of the Ramachandran plot show which values of the Phi/Psi angles are possible for an amino acid, X, in an ala-X-ala tripeptide (Wiltgen, 2019). The Ramachandran plot analysis of protein AChE showed high conformational quality, with no outliers identified. All 537 residues (100%) were in acceptable regions (>99.8%), with 96.6% (519/537) falling within favoured regions (>98%). The findings show the strong structural integrity of AChE (Sobolev et al., 2020).

Figure 1. The alignment between the RCSB PDB structure and the 3D predicted structure of the 4M0E protein is depicted. The resolved missing residues and the conservation of the protein structure compared to its actual PDB sequence are shown.

Virtual Screening with PyRx

Using PyRx 0.8 docking tools, the original phytochemicals, and four others with a similar structure were docked. The affinity of tacrine, donepezil, galantamine, and rivastigmine binding was considered as positive control which is -9.0 kcal/mol, -7.3 kcal/mol, -8.3 kcal/mol and -6.4 kcal/mol, and the value (kcal/mol) greater than that was considered as the target ligand. The primary screening was performed by compounds with greater binding affinity than tacrine, rivastigmine, donepezil, and galantamine. A total of 620 molecules have exhibited higher binding affinity than the control molecules (tacrine, donepezil, rivastigmine, and galantamine), including 18 phytochemicals sourced from the Dr. Dukes database (https://phytochem.nal.usda.gov/) (Table S4).

ADME profiling of Screened phytochemicals

The SwissADME (http://www.swissadme.ch/index.php) was utilized to examine the ADME profile and ability to traverse the blood-brain barrier for the selected 638 phytochemicals. During this phase of the investigation, most of the phytochemicals did not meet the drug-likeness property that was assessed. Lipinski’s rule states that, historically, 90% of orally absorbed drugs had fewer

than 5 H-bond donors, less than 10 H-bond acceptors, molecular weight of less than 500 Daltons and XlogP values of less than 5 (Dai et al., 2016). Due to their high solubility, many phytochemicals may struggle to penetrate the blood-brain barrier (BBB). Therefore, compounds with a blood-brain barrier permeability (BBB) equal to or higher than 0.477 (Log 3) were prioritized for analysis as potentially potent BBB-permeable candidates. Additionally, high gastrointestinal (GI) absorption was assessed. A comprehensive analysis of the ADME (absorption, distribution, metabolism, and excretion) and docking results for similar chemical and phytochemical structures was performed (Tables 2, 3, 4 and 5). These tables provide valuable insights into the compounds' pharmacokinetic properties and their potential interactions with target proteins. A total of 89 compounds along with phytochemicals were found to possess the properties that were assessed (Table S5).

Computational molecular docking with AutoDock

Outperforming control compounds tacrine, donepezil, galantamine, and rivastigmine, 88 identified molecules exhibit enhanced binding affinity in molecular docking via AutoDock Vina-1.5.7. These findings suggest their potential as promising acetylcholinesterase inhibitors, warranting further investigation, this study establishes a benchmark for assessing the comparative efficacy of the identified molecules with the positive control. The docking and redocking outcomes for the remaining compounds are comprehensively presented in the accompanying tables, encapsulating a comprehensive overview of their binding characteristics for further analytical consideration. This nuanced evaluation contributes to the burgeoning discourse surrounding potential therapeutic candidates for the development of novel acetylcholinesterase inhibitors (Motebennur et al., 2023). The binding affinities of rivastigmine analog compounds, which exhibit both blood-brain barrier (BBB) permeability and favorable drug-likeness characteristics, were further investigated (Table 2). Notably, three rivastigmine analogs, such as 10989924 ([3-(1-methylpiperidin-2-yl)phenyl] N,N-diethylcarbamate), 74817986 ([3-[1-[methyl(1-phenylethyl)amino]ethyl]phenyl] N-ethyl-N-methylcarbamate) and 46898202 [3-(1-piperidin-1-ylethyl)phenyl] N,N-diethylcarbamate , exhibited superior docking affinities as compared to rivastigmine. This observation suggests a potential enhancement in the binding interactions of these molecules with the target receptor.

Table 2. The docking, redocking and ADME results of Rivastigmine's similar structure with CID and chemical name.

Sl no	CID	IUPAC Name	Binding Affinity	Redocking	BBB	Rules 5 violation	GI absorption	Leadlikeness violations
1	77991	Rivastigmine	-6.4	-6.5	0.508	0	High	0
2	70266158	[2-[1-(azetidin-1-yl)ethyl]phenyl] N,N-dimethylcarbamate	-8.1	-7.3	0.564	0	High	2
3	66717459	[3-[(1S)-1-(dimethylamino)ethyl]-2-tritiophenyl] N-ethyl-N-methylcarbamate	-8.1	-7.9	0.506	0	High	1
4	42604975	[3-[(1S)-1-[methyl-[(1S)-1-phenylethyl]amino]ethyl]phenyl] N-ethyl-N-methylcarbamate	-8	-7.7	0.501	0	High	2
5	129309692	[3-[1-[[(1S)-1-cyclohexa-1,3-dien-1-ylethyl]-methylamino]ethyl]phenyl] N-ethyl-N-methylcarbamate	-7.9	-7.5	0.502	0	High	2
6	68377091	[3-[(1S)-1-(dimethylamino)ethyl]phenyl] N-ethynyl-N-[(2R)-1-phenylpropan-2-yl]carbamate	-7.7	-8.2	0.516	0	High	2
7	144066490	[3-[1-(dimethylamino)ethyl]phenyl] N-methyl-N-[(2R)-1-phenylpropan-2-yl]carbamate	-7.7	-7.7	0.506	0	High	3
8	10989924	[3-(1-methylpiperidin-2-yl)phenyl] N,N-diethylcarbamate	-7.6	-8.3	0.528	0	High	2
9	11359764	[3-[(1S)-1-[methyl(trideuterio(113C)methyl)amino]ethyl]phenyl] N-methyl-N-(1,1,2,2,2-pentadeuterio(213C)ethyl)carbamate	-7.6	-7.2	0.506	0	High	0
10	46898202	[3-(1-piperidin-1-ylethyl)phenyl] N,N-diethylcarbamate	-7.6	N/A	0.506	0	High	2
11	149047000	[3-[1-(dimethylamino)cyclopropyl]phenyl] N-ethyl-N-methylcarbamate	-7.6	-7.2	0.505	0	High	2
12	144474639	[3-[(1S)-1-[[(1S)-1-cyclohexa-2,4-dien-1-ylethyl]-methylamino]ethyl]phenyl] N-ethyl-N-methylcarbamate	-7.6	-7	0.501	0	High	0
13	21767521	7-[1-(dimethylamino)ethyl]-3-methyl-5,6-dihydro-4H-1,3-benzoxazocin-2-one	-7.5	-6.6	0.555	0	High	2
14	21767510	6-[1-(dimethylamino)ethyl]-3-methyl-4,5-dihydro-1,3-benzoxazepin-2-one	-7.4	-6.2	0.546	0	High	0
15	25204947	[3-[(1S)-1-(dimethylamino)ethyl]phenyl] N-methyl-N-[(2S)-1-phenylpropan-2-yl]carbamate	-7.4	-7.8	0.506	0	High	1
16	72816136	[3-[1-(dimethylamino)ethyl]phenyl] N-methyl-N-(1-phenylpropan-2-yl)carbamate	-7.4	-7.7	0.506	0	High	2
17	13955119	[2-[1-(dimethylamino)ethyl]phenyl] N,N-dimethylcarbamate	-7.3	-6.1	0.478	0	High	2
18	141557115	[3-[1-(dimethylamino)pentyl]phenyl] acetate	-7.3	-6.3	0.566	0	High	1
19	21767515	9-[1-(dimethylamino)ethyl]-3-methyl-5,6-dihydro-4H-1,3-benzoxazocin-2-one	-7.2	-7.3	0.547	0	High	1
20	21767496	5-[1-(dimethylamino)ethyl]-3-methyl-4H-1,3-benzoxazin-2-one	-7.2	-6.6	0.540	0	High	1
21	10935608	[2-(1-piperidin-1-ylethyl)phenyl] N,N-diethylcarbamate	-7.2	-7.2	0.520	0	High	0
22	10924256	[3-(piperidin-1-ylmethyl)phenyl] N,N-diethylcarbamate	-7.2	-7.2	0.517	0	High	1
23	144474633	[3-[(2S)-1-(dimethylamino)propan-2-yl]phenyl] N-ethyl-N-methylcarbamate	-7.1	-6.7	0.503	0	High	0
24	25230721	[3-[(1S)-1,2,2,2-tetradeuterio-1-(dimethylamino)ethyl]phenyl] N-ethyl-N-methylcarbamate	-7.1		0.509	0	High	1
25	51037855	[3-[(1S)-1,2,2,2-tetradeuterio-1-(dimethylamino)(213C)ethyl]phenyl] N-ethyl-N-methylcarbamate	-7.1	-6.4	0.508	0	High	0
26	51038065	[3-[(1S)-1-[methyl(trideuterio(113C)methyl)amino]ethyl]phenyl] N-ethyl-N-methylcarbamate	-7.1	-6.4	0.508	0	High	0

27	21767507	[3-[(1S)-1-[methyl(trideuterio(113C)methyl)amino]ethyl]phenyl] N-methyl-N-(1,1,2,2,2-pentadeuterio(213C)ethyl)carbamate	-7.1	-7.3	0.508	0	High	0
28	9823072	[3-[(1S)-1-(dimethylamino)ethyl]-2-tritiophenyl] N-ethyl-N-methylcarbamate	-7.1	-6.8	0.497	0	High	0
29	53705187	[2-[[ethyl(methyl)amino]methyl]phenyl] N,N-dimethylcarbamate	-7	-6.6	0.493	0	High	1
30	97357026	[3-[(1R)-1-(dimethylamino)ethyl]phenyl] N,N-diethylcarbamate	-7	-6.9	0.517	0	High	0
31	11066683	[3-(1-piperidin-1-ylethyl)phenyl] N,N-diethylcarbamate	-6.9	-7.2	0.493	0	High	1
32	25230725	[3-[(1S)-1-[bis(trideuteriomethyl)amino]-1,2,2,2-tetradeuterioethyl]-2,4,5,6-tetradeuteriophenyl] N-(1,1,2,2,2-pentadeuterioethyl)-N-(trideuteriomethyl)carbamate	-6.8	-6.4	0.517	0	High	0
33	144198864	(1S)-1-(3-methoxyphenyl)-N,N-dimethylpropan-1-amine	-6.7	-6.1	0.508	0	High	0
34	67474850	[3-[(1S)-1-(dimethylamino)ethyl]-4-fluorophenyl] N-ethyl-N-methylcarbamate	-6.7	-6.7	0.764	0	High	0
35	10999871	[3-(piperidin-1-ylmethyl)phenyl] N,N-dimethylcarbamate	-6.7	-7.5	0.545	0	High	1
36	10586926	[3-[(1S)-1-(dimethylamino)ethyl]-2-tritiophenyl] N-ethyl-N-methylcarbamate	-6.7	-6.5	0.533	0	High	0
37	71316042	[3-(1-piperidin-1-ylethyl)phenyl] N,N-diethylcarbamate	-6.7	-6.6	0.508	0	High	0
38	745584	[2-[(dimethylamino)methyl]phenyl] N,N-dimethylcarbamate	-6.6	-6.5	0.493	0	High	0
39	25230720	[2-deuterio-3-[(1S)-1-[dideuteriomethyl(methyl)amino]ethyl]phenyl] N-ethyl-N-methylcarbamate	-6.6	-7.2	0.532	0	High	0
40	25230723	[3-[(1S)-1-(dimethylamino)ethyl]phenyl] N-ethyl-N-(trideuteriomethyl)carbamate	-6.6	-7.3	0.508	0	High	1
41	25230724	[3-[(1S)-1-(dimethylamino)ethyl]phenyl] N-methyl-N-(1,1,2,2,2-pentadeuterioethyl)carbamate	-6.6	-7.3	0.508	0	High	0
42	51037853	[3-[(1S)-1,2,2,2-tetradeuterio-1-(dimethylamino)(113C)ethyl]phenyl] N-ethyl-N-methylcarbamate	-6.6	-7.4	0.508	0	High	0
43	51038067	[3-[(1S)-1-[methyl(trideuterio(113C)methyl)amino]ethyl]phenyl] N-methyl-N-(1,1,2,2,2-pentadeuterio(213C)ethyl)carbamate	-6.6	-6.8	0.508	0	High	0
44	77991	[3-(1-piperidin-1-ylethyl)phenyl] N,N-diethylcarbamate	-6.6	-6.4	0.508	0	High	0
45	92044359	[3-[(1R)-1-[bis(trideuteriomethyl)amino]ethyl]phenyl] N-ethyl-N-methylcarbamate	-6.6	-6.2	0.508	0	High	0

The binding affinities of tacrine and its structurally analogous exhibited the highest binding affinities in the entirety of the conducted docking study (Table 3). Notably, 2-naphthalen-2-ylquinolin-4-amine emerges as the most promising candidate, displaying a substantial binding affinity of -10.3 kcal/mol (PyRx) and -10.7 kcal/mol (AutoDock). The overall binding affinities observed collectively underscore the potential of these compounds for further exploration and development. Conversely, the galantamine similar structures presents only two compounds, and among them 4,14-dimethyl-11-oxa-4 azatetracyclo [8.7.1.01,12.06,18]octadeca-6(18),7,9,15-tetraen-9-ol was the best binding affinity with -8.4 and -7.9 kcal/mol, as the remaining analogs were judiciously excluded during primary virtual screening and ADME profiling (Table 4). This

stringent selection process aims to ensure structural and pharmacokinetic viability, contributing to a refined pool of candidates with enhanced potential for subsequent stages of drug development.

Table 3. The Docking and redocking results of tacrine's similar structures with CID and chemical name.

Sl no	CID	IUPAC Name	Affinity Pyrx Kcal/mol	Redocking Autodock Kcal/mol	BBB	Rules 5 violation	GI absorption	Leadlikeness violations
1	1935	Tacrine	-9.0	--8.8	0.316	1	High	1
2	18403988	2-naphthalen-2-ylquinolin-4-amine	-10.3	-10.7	0.565	0	High	2
3	149800	N-benzylacridin-9-amine	-9.9	-9.4	0.625	0	High	1
4	402658	12-azatetracyclo[9.8.0.02,7.013,18]nonadeca-1(19),2,4,6,11,13,15,17-octaen-19-amine	-9.9	-9	0.54	0	High	2
5	54474520	3-[2-(7-fluoroquinolin-2-yl)ethenyl]aniline	-9.8	-9	0.596	0	High	2
6	3438772	2-phenyl-4-pyrrolidin-1-ylquinoline	-9.7	-9.4	0.559	0	High	2
7	18934490	N-phenylacridin-1-amine	-9.7	-7.7	0.485	0	High	3
8	11492743	4-fluoro-2-(6-fluoro-4-methylquinolin-2-yl)aniline	-9.6	-6.9	0.602	0	High	2
9	69799851	4-Amino-2-styrylquinoline	9.5	10.1	0.577	0	High	1
10	129829335	10-sulfidoacridin-10-ium	-9.5	-9	0.708	0	High	0
11	164587579	2-benzyl-6-fluoroquinolin-4-amine	-9.5	N/A	0.692	0	High	2
12	130408026	2-(7-fluoro-2-phenylquinolin-3-yl)ethanamine	-9.5	-7.5	0.533	0	High	2
13	22395290	2-[(E)-2-phenylethenyl]quinolin-4-amine	-9.5	-9.6	0.521	0	High	0
14	69799851	2-(2-phenylethenyl)quinolin-4-amine	-9.5	-10.1	0.521	0	High	2
15	696663	12-azatetracyclo[9.8.0.02,7.013,18]nonadeca-1(19),2,4,6,11,13,15,17-octaen-19-amine	-9.5	-8.5	0.495	0	High	0
16	402666	19-azatetracyclo[9.8.0.02,7.013,18]nonadeca-1(19),2,4,6,11,13,15,17-octaen-12-amine	-9.5	-9.9	0.483	0	High	1
17	10587156	6-fluoro-2-(2-fluorophenyl)quinolin-4-amine	-9.4	-9.8	0.692	0	High	2
18	1504001	2-phenyl-4-piperidin-1-ylquinoline	-9.4	-10	0.535	0	High	2
19	164587580	2-(2-fluorophenyl)quinolin-4-amine	-9.3	-8.2	0.662	0	High	1
20	60598	9-(4-methylpiperidin-1-yl)-1,2,3,4-tetrahydroacridine	-9.3	-9.1	0.596	0	High	1
21	4452632	3-quinolin-2-ylaniline	-9.3	-9.6	0.506	0	High	1
22	7742109	(NZ)-N-(1-phenyl-2-quinolin-2-ylethylidene)hydroxylamine	-9.3	-9.2	0.487	0	High	0
23	12102730	2,4-dimethylbenzo[h]quinolin-10-amine	-9.3	-9.7	0.48	0	High	1
24	21998	10-methylacridin-10-ium-9-amine	-9.2	-9.5	0.71	0	High	0
25	45599224	12-azatetracyclo[9.8.0.02,7.013,18]nonadeca-1(19),2,4,6,11,13,15,17-octaen-19-amine	-9.2	-8	0.653	0	High	1
26	45599463	5,7-difluoro-2-phenylquinolin-4-amine	-9.2	-9.7	0.637	0	High	0
27	22334541	N-(3-fluorophenyl)-2,3-dihydro-1H-cyclopenta[b]quinolin-9-amine	-9.2	-9.2	0.635	0	High	0
28	11737199	2-(2-fluorophenyl)quinolin-4-amine	-9.2	-6.7	0.583	0	High	0
29	55045454	6-methyl-2-phenylquinolin-4-amine	-9.2		0.484	0	High	0
30	31633	10-methylacridin-10-ium-3-amine	-9.1	-9.4	0.71	0	High	1
31	45599470	7,8-difluoro-2-phenylquinolin-4-amine	-9.1	-9.8	0.701	0	High	0

32	45599222	6-fluoro-2-phenylquinolin-4-amine	-9.1	-9.5	0.662	0	High	1
33	21828278	2,6-diphenylpyridin-4-amine	-9.1	-9.7	0.613	0	High	0
34	21639083	12-azatetracyclo[9.8.0.02,7.013,18]nonadeca-1(19),2,4,6,11,13,15,17-octaen-19-amine	-9.1	-7.4	0.607	0	High	0
35	43419931	N-[(4-fluorophenyl)methyl]-2-methylquinolin-4-amine	-9.1	-7.1	0.545	0	High	0
36	129641425	2-(2-phenylethenyl)quinolin-3-amine	-9.1	-9.2	0.521	0	High	1
37	12394207	2-phenyl-4-piperidin-1-ylquinoline	-9.1	-8.7	0.518	0	High	0
38	10980245	2-(2-fluorophenyl)quinolin-4-amine	-9.1	-8.6	0.506	0	High	0

Table 4. The Docking and redocking results of galantamine similar structure with CID and chemical name.

Sl no	CID (galantamine similar structures)	IUPAC Name	Affinity Pyrx Kcal/mol	Redocking Autodock Kcal/mol	BBB	Rules 5 violation	GI Absorption	Leadlikeness violations
1	9651	Galantamine	-8.3	-8.7	-0.08	0	High	0
2	91042094	9-methoxy-4-prop-2-enyl-11-oxa-4-azatetracyclo[8.6.1.01,12.06,17]heptadeca-6(17),7,9,15-tetraene	-8.6	-9.2	0.48	0	High	1
3	20706288	4,14-dimethyl-11-oxa-4 azatetracyclo[8.7.1.01,12.06,18]octadeca-6(18),7,9,15-tetraen-9-ol	-8.4	-7.9	0.59	0	High	0

Phytochemicals meeting the criteria of the blood-brain barrier (BBB) permeability and favourable drug-likeness were subjected to further investigation through molecular docking (Table 5). Among these, berberine exhibited a notable binding affinity of -9.3 kcal/mol, huperzine B demonstrated -8.3 kcal/mol, bisdemethoxycurcumin revealed -9.3 kcal/mol, and curcumin displayed a binding affinity of -9.2 kcal/mol. These findings highlight the substantial potential of these phytochemicals as candidates for acetylcholinesterase inhibition.

Table 5. The Docking results of phytochemicals with CID and chemical name.

Sl no	Ligand CID	IUPAC Name	Affinity Pyrx Kcal/mol	Redocking Autodock Kcal/mol	BBB	Rules 5 violation	GI Absorption	Leadlikeness violations
1	2353	Berberine	-9.3	-9.5	0.198	0	High	1
2	5315472	Bisdemethoxycurcumin	-9.3	-9.7	0.398	0	high	0
3	6916252	Huperzine B	-8.3	-8.4	0.489	0	High	0
4	854026	Huperzine A	-7.9	-7.5	0.317	0	High	1
5	160512	Ar-Turmerone	-7.6	-7.8	0.105	1	High	2
6	1253	(-)-Selagine	-6.9	-6.8	0.512	0	High	1

Docking site analysis

To conduct a more comprehensive investigation, a total of eight compounds (Table 6) have been chosen for a molecular dynamics (MD) simulation lasting 100 nanoseconds Based on the docking analysis and ADME profiling. Utilizing BioVia Discovery Studio, it is feasible to visually observe

the interaction between protein ligands and active site residues, as well as to overlay all proteins and ligands, based on their highest binding affinity and respective segments. The common residues involved in the positive controls tacrine, galantamine, rivastigmine, and donepezil are- Tyr340, Phe296, Trp285, Phe337, and Tyr123, and there was Tyr123 with a hydrogen bond and Trp285, Tyr340, and Phe296 with Pi-allyl interaction. However, the residues involved in the interaction and the binding sites exhibit similarities, as do the bonding characteristics. This suggests that the binding location and residues are congruent to those to which tacrine, donepezil rivastigmine galantamine bind.

Table 6. Docking site analysis for selected chemicals.

Sl no	Ligand name	Complex	Pubchem CID	Pyrx Docking	Autodock docking	Interacting Residues
1	[3-(1-methylpiperidin-2-yl)phenyl] N,N-diethylcarbamate	Complex_1	10989924	-7.6	-8.3	Tyr123, Tyr336, Tyr340, Phe337, Trp285
2	2-naphthalen-2-ylquinolin-4-amine	Complex_2	18403988	-10.3	-10.7	Tyr123, Tyr285, Tyr340, His286, Asp73
3	4-Amino-2-styrylquinoline	Complex_3	69799851	-9.5	-10.1	Tyr123, Phe337, Tyr336, Trp285, Trp85, Gly119, Gly120
4	9-methoxy-4-prop-2-enyl-11-oxa-4-azatetracyclo[8.6.1.01,12.06,17] heptadeca-6(17),7,9,15-tetraene	Complex_4	91042094	-8.6	-9.2	Leu288, Leu75, Phe337, Phe296, Tyr340, Trp285
5	Huperzine B	Complex_5	6916252	-	-8.3	Trp285, Tyr123, Tyr71, Leu71
6	Bisdemethoxycurcumin	Complex_6	5315472	-	-9.3	Trp285, Tyr340, Trp85, Tyr71, His446
7	Berberine	Complex_7	2353	-	-9.3	Tyr123, Tyr336, Tyr340, Phe337, Trp285, Ser292, His286
8	Ar-Turmerone	Complex_8	160512	-	-7.6	Tyr123, Tyr336, Tyr340, Phe337, Trp285, Phe296, Leu288

The 2D interaction analysis elucidates the nature of binding interactions (Fig. 2), revealing the presence of pi-alkyl and pi-sigma interactions while notably excluding electrostatic bonds. Notably, TYR123 exhibits hydrogen bonding, and TRP285 displays pi-alkyl interaction across all complexes. These residue interactions demonstrate a consistent pattern, underscoring the reproducibility of specific binding motifs within the studied complexes.

Figure 2: A visual representation of Protein-ligand interaction. The protein-ligand interaction of Complex_1 (A), Complex_2 (B), Complex_3 (C), Complex_4 (D), Complex_5 (E), Complex_6 (F), Complex_7 (G), and Complex_8 (H). All the interactions have common Tyr123 with a hydrogen bond and Trp85 with Pi-allyl interaction. The rest of the interactions have Pi-sigma with similar residues of the active side.

Molecular Dynamics Simulation analysis

The simulation was performed in a Desmond environment. There were 8 compounds primarily selected for MD simulation in the Desmond simulation environment. The overall simulation results were interpreted in RMSD, RMSF, Ligand properties, DCCM and PCA values. The binding grooves (Fig. 1) of the examined chemicals were superimposed, revealing a remarkable degree of similarity in their spatial arrangements. Additionally, the residues involved in interactions exhibited striking congruence among the compounds. This congruency in binding grooves and interacting residues suggests a conserved mode of binding, reinforcing the likelihood of a shared molecular mechanism or target engagement.

Figure 3: A visual representation of the binding pocket and ligand interaction. (A) The 3d Structure of protein-ligand complex and protein hydrophobicity mapping. Close view of Complex_1 (B), Complex_3 (C), Complex_6 (D). The protein pocket region is slightly bluish which indicates partially hydrophilic. All the ligands bind to the same side of the protein.

The RMSD of Protein-ligand Complex figures have shown the Protein RMSD fit with ligand RMSD over a 100ns time scale. RMSD, which is the ligand insect in the protein RMSD line considered a good stability benchmark. Complex_1 Complex_3 and Complex_6 show better binding stability (Fig. 3). The Root Mean Square Fluctuation (RMSF) is a valuable tool for quantifying localized variations along the protein chain. Peaks on the plots represent regions of the protein that exhibit the highest degree of fluctuation throughout the simulation. It is commonly observed that the tails, specifically the N- and C-terminal, exhibit greater fluctuations compared to other regions of the protein. Secondary structure elements, such as alpha helices and beta strands, typically exhibit greater rigidity compared to the unstructured regions of the protein. As a result, they undergo less fluctuation than the loop regions (Fig. 4).

Figure 4: A 100-nanosecond simulation is conducted to measure the root mean square deviation (RMSD). Results of four complexes. Complexes 1, 2, and 3 are subjected to a 100-nanosecond molecular dynamics simulation using the Desmond software. A) RMSD of Complex_1. B) RMSD of Complex_3. C) RMSD of Complex_6. The root means square deviation (RMSD) between the ligand and protein exhibits temporal constancy, thereby ensuring stability.

Nevertheless, complex_1 and 3 demonstrate persistent stability, suggesting that the interaction between the protein and ligand remains intact throughout the entire duration. Complex_6 exhibits a deviation of 30ns, indicating inferior stability compared to the other 2 complexes. Nevertheless, the overall binding interaction is not significantly unfavourable, and further investigation is required for the other parameters.

A ligand exhibiting a moderate degree of compactness, as measured by a moderate gyration value, could potentially achieve a harmonious equilibrium between sufficient molecular surface area (SASA) for interaction purposes and accessibility for binding. The combination of moderate gyration and a larger molecular surface area may provide numerous binding interaction sites, whereas a moderate SASA may indicate a stable structure with restricted solvent exposure (Fig. 5).

Figure 5: The root means square fluctuation (RMSF) of all the simulation complexes over a 100-nanosecond simulation. A- Root Mean Square Fluctuation (RMSF) of Complex_1, B- RMSF of Complex_3, C- RMSF of Complex_6. The interpretation of the results is justified. Several significant fluctuations. The fluctuation primarily arises when the ligand interacts with the protein residues. Complex_1 exhibits three significant fluctuations on the green vertical bar, which signify the contact between the ligand molecule and the protein. Complex_3 and Complex_6 exhibit significant temporal fluctuations. The overall comparison reveals significant fluctuations, although they do not exceed 4.8 Å.

The gyration results indicate that Complex_1 and Complex_3 is located within a range of 3.5-4.00 Armstrong, while Complex_6 is situated between 5.0-5.5 Å (Fig. 6A). A higher value of the radius of gyration indicates a greater dispersion of atoms and a longer molecule. This metric quantifies the degree of elongation of a ligand and is equal to its primary moment of inertia. The SASA analysis reveals superior ligand characteristics, specifically in Complex_3 and Complex_6, with a surface area ranging from 50 to 100 Armstrong square units (Fig. 6B). Reduced solvent-accessible surface area (SASA) leads to increased binding stability. The polar surface area and the molecular surface area exhibit significant differences. Complex_1 exhibits lower levels of PSA and higher levels of MolSA, whereas Complex_6 displays higher levels of both PSA and MolSA (Fig. 6, C

and D). Complex_6 exhibits reduced levels of PSA and MolSA. Elevated PSA levels can potentially impact binding employing electrostatic interactions. A greater MolSA value signifies an increased number of sites available for interacting with other molecules or receptors.

Figure 6: A 100ns simulation of Ligand Properties of all the Complexes. (A) Ligand Gyration, (B) Ligand SASA, (C) Ligand Polar Surface Area (PSA), and (D) Molecular Surface Area (MolSA). Values of complex_1, complex_3, and complex_6 are represented with blue, orange, and green colour, respectively.

PCA analysis

Principal Component Analysis (PCA) is a mathematical technique that identifies the most significant components in a dataset by analyzing the covariance or correlation matrix. In the context of protein analysis, PCA utilizes atomic coordinates to define the protein's available degrees of freedom (DOF). The result of those three results PCAs has been performed (Fig. 7). PCA analysis of each of the component percentage indicate each of the parameters, PC1 might indicate how strongly the ligand binds to the protein, PC2 could represent something like the flexibility of the protein-ligand complex and PC3 might capture variations in the shape complementarity between the protein and ligand.

Figure 7: PCA analysis of Three Complexes. The PCA of Complex_1 (A), complex_2 (B), and complex_3 (C). The White dot here mentioning the transition state of protein ligand simulation confirmation, the blue dot with a scattered indicates energetically unstable conformational states and red dots indicate the stable conformational state.

The highest percentage of variance explained is indicated by the Single Component with the Highest Variance (PC1), as determined by the PCA analysis. Complex_1 PCA yields the most favourable outcomes, followed by complex_3 and complex_6. By considering the amalgamation of constituents that capture substantial variation in contrast to the summaries of 46.18% and 41.54% for both complexes, Complex_1 exhibits a sum of 53% (Table 7). It exhibits improved variances. Complex 1 exhibits superior performance in both analyses, whether a singular

component with the highest variance is considered or a collection of components that collectively account for a substantial proportion of the data's variance is considered (David & Jacobs, 2014a).

Table 7. Different PCA components chart of each of the complexes

Complex	PCA Components		
	PC1 (%)	PC2 (%)	PC3 (%)
Complex_1	44.7	8.21	6.76
Complex_3	35.3	10.88	7.01
Complex_6	33.21	8.33	5.75

DCCM analysis

The DCCM analysis method was applied in a novel way to assist in the identification of potential protein domains. During the implementation of this novel approach, multiple DCCM maps were computed, each utilizing a distinct coordinate reference frame to determine the boundaries of protein domains and the constituents of protein domain residues (Nascimento et al., 2022).

Figure 8: The cross-correlation map of the C α atom pairs within the monomers of AChE is analyzed for dynamics. The DCCM of Complex_1 (A), complex_2 (B), and complex_3 (C). The correlation coefficient (C ij) was represented using various colours. The values of Cij, ranging from 0 to 1, indicate positive correlations. Positive correlations indicate that these pairs of atoms tend to move in similar directions or have comparable behaviours during the simulation. On the other hand, negative correlations are represented by Cij values ranging from -1 to 0. Negative correlations indicate that these pairs of atoms tend to migrate in opposite directions or have contrasting behaviours during the simulation.

Discussion

The therapeutic intervention of Alzheimer's disease (AD) using acetylcholinesterase inhibitors (AChEi) has been demonstrated by a wide range of plant-based compounds (Santos et al., 2018). Given the absence of reliable, efficient, and secure inhibitors, investigating structurally similar compounds could be a promising field for researchers to explore (Čolović et al., 2013). In this study, we analyzed the chemical structures of tacrine, donepazel, galantamine, and rivastigmine to identify potential alternative drugs that are safer (Ahmed et al., 2021). Computer aid drug design (CADD) methodologies have been discovered to expand the repositories of chemical compounds

for the identification of potential inhibitors. The assessment of the binding affinity between a protein and a vast collection of ligands is frequently accomplished through the application of molecular docking techniques (Baig et al., 2018). The molecules within the applicability domain of the constructed-in silico model were screened to assess their drug-likeness and ADME properties. Drug likeness provides a highly valuable criterion for determining the minimum requirements that a compound must meet to be considered suitable for drug development (Gleeson et al., n.d.). This criterion helps in the objective selection of new drug candidates that have desirable bioavailability (Hefti, 2008).

Molecular docking is a highly effective approach in CADD that utilizes specific algorithms to determine the affinity scores based on the positioning of ligands within the binding pocket of a target. In molecular docking, the lowest docking score corresponds to the highest affinity, indicating that the complex remains in contact for a longer period with good stability (Agu et al., 2023; Meng et al., 2011). Rigorously examine the protein-ligand binding to identify compounds with higher binding affinity and potentially improved hydrogen bonding characteristics (Du et al., 2016). The analysis of the docking results confirmed the binding of the final three compounds, including [3-(10methylpiperidin-2-yl)phenyl]. The residues Tyr123, Tyr336, Tyr340, Phe337, and Trp285 are involved in the interaction with N,N-diethyl carbamate. Specifically, Compound 3, identified as 4-amino-2-styrylquinoline, interacts with the residues Tyr 123, Phe 337, Tyr 336, Trp 285, Trp 85, Gly 119, and Gly 120. Conversely, Compound 6, known as Bisdemethoxycurcumin, binds to the residues Trp 285, Tyr 123, Trp 85, Tyr 71, and His 446.

Molecular dynamics simulations demonstrated stable interactions between specific ligands and the AChE binding site. Notably, compounds like [3-(1-methylpiperidin-2-yl)phenyl] N,N-diethylcarbamate, 4-Amino-2-styrylquinoline and Bisdemethoxycurcumin displayed consistent and favorable interactions throughout the simulation period. Such stability suggests a potential for these compounds to serve as stable and effective inhibitors. The RMSD and RMSF values of these complexes remained quite stable throughout the simulation. Specifically, the complex involving 4-Amino-2-styrylquinoline exhibited stability with a constant value over time. Similarly, [3-(1-methylpiperidin-2-yl)phenyl] N,N-diethylcarbamate also demonstrated stability during the simulation. Although the RMSD of Bisdemethoxycurcumin deviated, indicating a slight variation in the protein-ligand fit, the overall stability remained satisfactory. PCA and DCCM analysis of those three compounds were performed. Principal Component Analysis (PCA) in molecular

dynamics studies elucidates key factors influencing protein-ligand interactions. PC1 signifies binding strength, PC2 reflects protein-ligand complex flexibility, and PC3 captures shape complementarity. Higher PC1 scores denote stronger interactions, while elevated PC2 scores suggest increased complex flexibility. Enhanced PC3 scores indicate superior geometric fit between protein and ligand (David & Jacobs, 2014b). Complex_1, comprising [3-(1-methylpiperidin-2-yl) phenyl] N, N-diethyl carbamate, binds with AChE and demonstrates superior performance in PC analysis. Additionally, Compounds 3 (4-Amino-2-styrylquinoline) and 6 (Bisdemethoxycurcumin) exhibit promising results in PCA. Conversely, the DCCM analysis of compound 1 reveals a positive correlation among the protein-protein residues throughout the simulation, alongside stable correlations with certain compounds exhibiting both positive and negative associations (Avti et al., 2022).

Exploring the potential of computationally screened compounds in comparison to established drugs for Alzheimer's disease shows a promising direction for future research (Ahmed et al., 2021). Experimental validation using in vitro and in vivo studies is essential to confirm the effectiveness and safety characteristics of these identified compounds. Recognizing the constraints of the computational approach is crucial, including the inherent approximations in modelling, the possibility of false positives, and the requirement for experimental verification. The intricate characteristics of AD pathophysiology pose difficulties in identifying specific inhibitors that efficiently target the progression of the disease (Golriz Khatami et al., 2020). The combination of computational screening and molecular dynamics simulations provides an initial yet insightful view on potential inhibitors for AD (Lemkul & Bevan, 2012). The identified compounds show potential as candidates for further investigation and confirmation in preclinical and clinical studies. Nevertheless, the practical application of these compounds as effective treatments necessitates thorough experimental verification (Siddiqui et al., 2017).

Conclusion

The treatment of Alzheimer's disease through acetylcholinesterase inhibitors has been showcased by various plant-derived compounds. Considering the scarcity of dependable, effective, and safe inhibitors, exploring compounds with comparable structures holds promise as a potential avenue for investigation. One of the quickest and most economical methods is computational techniques. Computational biology has shown that different types of chemicals from plants and marine sources

have been identified and found to possess strong inhibitory effects against cholinesterase. In this study, we performed a virtual screening to discover new cholinesterase inhibitors from similar structures and plant compounds that interact with cholinesterase. Docking and molecular simulation tools were employed to investigate the significance of binding interactions of potentially new molecules for Alzheimer's disease treatment.

Data Availability

All data supporting the described findings of the study can be obtained from the corresponding authors upon request.

Funding

The authors declare that no funds, grants, or other support were received during the preparation of this manuscript.

Competing Interests

The authors have no relevant financial or non-financial interests to disclose.

Authors' Contributions

Mahir Azmal performed experiments, analyzed data, and wrote the draft. Md. Sahadot Hossen, Naimul Haque Shohan, and Md Rasid Taqui performed the experiments and analyzed the data. Abbeha Malik performed the simulation experiment and analyzed the data. Ajit Ghosh conceived and designed the experiments. All the authors read the final version of the manuscript.

Ethics declarations

Ethics Approval

Not applicable.

Consent for Publication

Not applicable.

Acknowledgement

The authors acknowledge the logistic support and laboratory facilities of the Department of Biochemistry and Molecular Biology, Shahjalal University of Science and Technology, Sylhet, Bangladesh.

References

Agu, P. C., Afiukwa, C. A., Orji, O. U., Ezeh, E. M., Ofoke, I. H., Ogbu, C. O., Ugwuja, E. I., & Aja, P. M. (2023). Molecular docking as a tool for the discovery of molecular targets of nutraceuticals in diseases management. *Scientific Reports*, *13*(1), Article 1. https://doi.org/10.1038/s41598-023-40160-2

Ahmed, S., Khan, S. T., Zargaham, M. K., Khan, A. U., Khan, S., Hussain, A., Uddin, J., Khan, A., & Al-Harrasi, A. (2021). Potential therapeutic natural products against Alzheimer's disease with Reference of Acetylcholinesterase. *Biomedicine & Pharmacotherapy*, *139*, 111609. https://doi.org/10.1016/j.biopha.2021.111609

Anand, P., Singh, B., & Singh, N. (2012). A review on coumarins as acetylcholinesterase inhibitors for Alzheimer's disease. *Bioorganic & Medicinal Chemistry*, *20*(3), 1175–1180. https://doi.org/10.1016/j.bmc.2011.12.042

Avti, P., Chauhan, A., Shekhar, N., Prajapat, M., Sarma, P., Kaur, H., Bhattacharyya, A., Kumar, S., Prakash, A., Sharma, S., & Medhi, B. (2022). Computational basis of SARS-CoV 2 main protease inhibition: An insight from molecular dynamics simulation based findings. *Journal of Biomolecular Structure and Dynamics*, *40*(19), 8894–8904. https://doi.org/10.1080/07391102.2021.1922310

Baig, M. H., Ahmad, K., Rabbani, G., Danishuddin, M., & Choi, I. (2018). Computer Aided Drug Design and its Application to the Development of Potential Drugs for Neurodegenerative Disorders. *Current Neuropharmacology*, *16*(6), 740–748. https://doi.org/10.2174/1570159X15666171016163510

Bartolucci, C., Perola, E., Pilger, C., Fels, G., & Lamba, D. (2001). Three-dimensional structure of a complex of galanthamine (Nivalin®) with acetylcholinesterase from Torpedo californica: Implications for the design of new anti-Alzheimer drugs. *Proteins: Structure, Function, and*

Bioinformatics, *42*(2), 182–191. https://doi.org/10.1002/1097-0134(20010201)42:2<182::AID-PROT50>3.0.CO;2-1

Birks, J. S., & Harvey, R. (2003). Donepezil for dementia due to Alzheimer's disease. *The Cochrane Database of Systematic Reviews*, *3*, CD001190. https://doi.org/10.1002/14651858.CD001190

Breijyeh, Z., & Karaman, R. (2020). Comprehensive Review on Alzheimer's Disease: Causes and Treatment. *Molecules*, *25*(24), Article 24. https://doi.org/10.3390/molecules25245789

Čolović, M. B., Krstić, D. Z., Lazarević-Pašti, T. D., Bondžić, A. M., & Vasić, V. M. (2013). Acetylcholinesterase Inhibitors: Pharmacology and Toxicology. *Current Neuropharmacology*, *11*(3), 315–335. https://doi.org/10.2174/1570159X11311030006

Dai, S.-X., Li, W.-X., Han, F.-F., Guo, Y.-C., Zheng, J.-J., Liu, J.-Q., Wang, Q., Gao, Y.-D., Li, G.-H., & Huang, J.-F. (2016). In silico identification of anti-cancer compounds and plants from traditional Chinese medicine database. *Scientific Reports*, *6*. https://doi.org/10.1038/srep25462

Daina, A., Michielin, O., & Zoete, V. (2017). SwissADME: A free web tool to evaluate pharmacokinetics, drug-likeness and medicinal chemistry friendliness of small molecules. *Scientific Reports*, *7*, 42717. https://doi.org/10.1038/srep42717

Dallakyan, S., & Olson, A. J. (2015a). Small-molecule library screening by docking with PyRx. *Methods in Molecular Biology (Clifton, N.J.)*, *1263*, 243–250. https://doi.org/10.1007/978-1-4939-2269-7_19

Dallakyan, S., & Olson, A. J. (2015b). Small-molecule library screening by docking with PyRx. *Methods in Molecular Biology (Clifton, N.J.)*, *1263*, 243–250. https://doi.org/10.1007/978-1-4939-2269-7_19

David, C. C., & Jacobs, D. J. (2014a). Principal Component Analysis: A Method for Determining the Essential Dynamics of Proteins. *Methods in Molecular Biology (Clifton, N.J.)*, *1084*, 193–226. https://doi.org/10.1007/978-1-62703-658-0_11

David, C. C., & Jacobs, D. J. (2014b). Principal Component Analysis: A Method for Determining the Essential Dynamics of Proteins. *Methods in Molecular Biology (Clifton, N.J.)*, *1084*, 193–226. https://doi.org/10.1007/978-1-62703-658-0_11

dos Santos Nascimento, I. J., De Souza, M., Medeiros, D. C., & de Moura, R. O. (2022). Dynamic Cross-Correlation Matrix (DCCM) Reveals New Insights to Discover New NLRP3 Inhibitors Useful as Anti-Inflammatory Drugs. *Medical Sciences Forum*, *14*(1), Article 1. https://doi.org/10.3390/ECMC2022-13306

Du, X., Li, Y., Xia, Y.-L., Ai, S.-M., Liang, J., Sang, P., Ji, X.-L., & Liu, S.-Q. (2016). Insights into Protein–Ligand Interactions: Mechanisms, Models, and Methods. *International Journal of Molecular Sciences*, *17*(2), 144. https://doi.org/10.3390/ijms17020144

Du, X., Wang, X., & Geng, M. (2018). Alzheimer's disease hypothesis and related therapies. *Translational Neurodegeneration*, *7*(1), 2. https://doi.org/10.1186/s40035-018-0107-y

Gleeson, M. P., Hersey, A., & Hannongbua, S. (n.d.). In-Silico ADME Models: A General Assessment of their Utility in Drug Discovery Applications. *Current Topics in Medicinal Chemistry*, *11*(4), 358–381.

Golriz Khatami, S., Robinson, C., Birkenbihl, C., Domingo-Fernández, D., Hoyt, C. T., & Hofmann-Apitius, M. (2020). Challenges of Integrative Disease Modeling in Alzheimer's Disease. *Frontiers in Molecular Biosciences*, *6*, 158. https://doi.org/10.3389/fmolb.2019.00158

Hefti, F. F. (2008). Requirements for a lead compound to become a clinical candidate. *BMC Neuroscience*, *9*(Suppl 3), S7. https://doi.org/10.1186/1471-2202-9-S3-S7

Kim, J., Lee, H. J., & Lee, K. W. (2010). Naturally occurring phytochemicals for the prevention of Alzheimer's disease. *Journal of Neurochemistry*, *112*(6), 1415–1430. https://doi.org/10.1111/j.1471-4159.2009.06562.x

Kuntz, I. D. (1992). Structure-Based Strategies for Drug Design and Discovery. *Science*, *257*(5073), 1078–1082. https://doi.org/10.1126/science.257.5073.1078

Lagadic-Gossmann, D., Rissel, M., Le Bot, M. A., & Guillouzo, A. (1998). Toxic Effects of Tacrine on Primary Hepatocytes and Liver Epithelial Cells in Culture. *Cell Biology and Toxicology*, *14*(5), 361–373. https://doi.org/10.1023/A:1007589808761

Lemkul, J. A., & Bevan, D. R. (2012). The Role of Molecular Simulations in the Development of Inhibitors of Amyloid β-Peptide Aggregation for the Treatment of Alzheimer's Disease. *ACS Chemical Neuroscience*, *3*(11), 845–856. https://doi.org/10.1021/cn300091a

Lopa, S. S., Al-Amin, Md. Y., Hasan, Md. K., Ahammed, Md. S., Islam, K. M., Alam, A. H. M. K., Tanaka, T., & Sadik, Md. G. (2021). Phytochemical Analysis and Cholinesterase Inhibitory and Antioxidant Activities of Enhydra fluctuans Relevant in the Management of Alzheimer's Disease. *International Journal of Food Science*, *2021*, 8862025. https://doi.org/10.1155/2021/8862025

Malik, A., Iqbal, M. N., Ashraf, S., Khan, M. S., Shahzadi, S., Shafique, M. F., Sajid, Z., Sajid, M., & Sehgal, S. A. (2023). In silico elucidation of potential drug targets against oxygenase domain of Human eNOS Dysfunction. *PLOS ONE*, *18*(4), e0284993. https://doi.org/10.1371/journal.pone.0284993

Meng, X.-Y., Zhang, H.-X., Mezei, M., & Cui, M. (2011). Molecular Docking: A powerful approach for structure-based drug discovery. *Current Computer-Aided Drug Design*, *7*(2), 146–157.

Motebennur, S. L., Nandeshwarappa, B. P., & Katagi, M. S. (2023). Drug Candidates for the Treatment of Alzheimer's Disease: New Findings from 2021 and 2022. *Drugs and Drug Candidates*, *2*(3), Article 3. https://doi.org/10.3390/ddc2030030

Olin, J., & Schneider, L. (2002). Galantamine for Alzheimer's disease. *The Cochrane Database of Systematic Reviews*, *3*, CD001747. https://doi.org/10.1002/14651858.CD001747

Onor, M. L., Trevisiol, M., & Aguglia, E. (2007). Rivastigmine in the treatment of Alzheimer's disease: An update. *Clinical Interventions in Aging*, *2*(1), 17–32. https://doi.org/10.2147/ciia.2007.2.1.17

Pilger, C., Bartolucci, C., Lamba, D., Tropsha, A., & Fels, G. (2001). Accurate prediction of the bound conformation of galanthamine in the active site of torpedo californica acetylcholinesterase using molecular docking11Color Plates for this article are on pages 374–378. *Journal of Molecular Graphics and Modelling*, *19*(3), 288–296. https://doi.org/10.1016/S1093-3263(00)00056-5

Rathod, S., Shinde, K., Porlekar, J., Choudhari, P., Dhavale, R., Mahuli, D., Tamboli, Y., Bhatia, M., Haval, K. P., Al-Sehemi, A. G., & Pannipara, M. (2023). Computational Exploration of Anti-cancer Potential of Flavonoids against Cyclin-Dependent Kinase 8: An *In Silico* Molecular Docking and Dynamic Approach. *ACS Omega*, *8*(1), 391–409. https://doi.org/10.1021/acsomega.2c04837

Roberson, M. R., & Harrell, L. E. (1997). Cholinergic activity and amyloid precursor protein metabolism. *Brain Research. Brain Research Reviews*, *25*(1), 50–69. https://doi.org/10.1016/s0165-0173(97)00016-7

Santos, T. C. dos, Gomes, T. M., Pinto, B. A. S., Camara, A. L., & Paes, A. M. de A. (2018). Naturally Occurring Acetylcholinesterase Inhibitors and Their Potential Use for Alzheimer's Disease Therapy. *Frontiers in Pharmacology*, *9*. https://www.frontiersin.org/journals/pharmacology/articles/10.3389/fphar.2018.01192

Sarkar, B., Alam, S., Rajib, T. K., Islam, S. S., Araf, Y., & Ullah, Md. A. (2021). Identification of the most potent acetylcholinesterase inhibitors from plants for possible treatment of Alzheimer's disease: A computational approach. *Egyptian Journal of Medical Human Genetics*, *22*(1), 10. https://doi.org/10.1186/s43042-020-00127-8

Scheltens, P., Strooper, B. D., Kivipelto, M., Holstege, H., Chételat, G., Teunissen, C. E., Cummings, J., & Flier, W. M. van der. (2021). Alzheimer's disease. *The Lancet*, *397*(10284), 1577–1590. https://doi.org/10.1016/S0140-6736(20)32205-4

Siddiqui, M. R., AlOthman, Z. A., & Rahman, N. (2017). Analytical techniques in pharmaceutical analysis: A review. *Arabian Journal of Chemistry*, *10*, S1409–S1421. https://doi.org/10.1016/j.arabjc.2013.04.016

Sobolev, O. V., Afonine, P. V., Moriarty, N. W., Hekkelman, M. L., Joosten, R. P., Perrakis, A., & Adams, P. D. (2020). A Global Ramachandran Score Identifies Protein Structures with Unlikely Stereochemistry. *Structure*, *28*(11), 1249-1258.e2. https://doi.org/10.1016/j.str.2020.08.005

Taqui, R., Debnath, M., Ahmed, S., & Ghosh, A. (2022). Advances on plant extracts and phytocompounds with acetylcholinesterase inhibition activity for possible treatment of Alzheimer's disease. *Phytomedicine Plus*, *2*(1), 100184. https://doi.org/10.1016/j.phyplu.2021.100184

Tayeb, H. O., Yang, H. D., Price, B. H., & Tarazi, F. I. (2012). Pharmacotherapies for Alzheimer's disease: Beyond cholinesterase inhibitors. *Pharmacology & Therapeutics*, *134*(1), 8–25. https://doi.org/10.1016/j.pharmthera.2011.12.002

Wiltgen, M. (2019). Algorithms for Structure Comparison and Analysis: Homology Modelling of Proteins. In S. Ranganathan, M. Gribskov, K. Nakai, & C. Schönbach (Eds.), *Encyclopedia of Bioinformatics and Computational Biology* (pp. 38–61). Academic Press. https://doi.org/10.1016/B978-0-12-809633-8.20484-6

Figure 1

Click here to access/download;Figure;Figure 1.tif

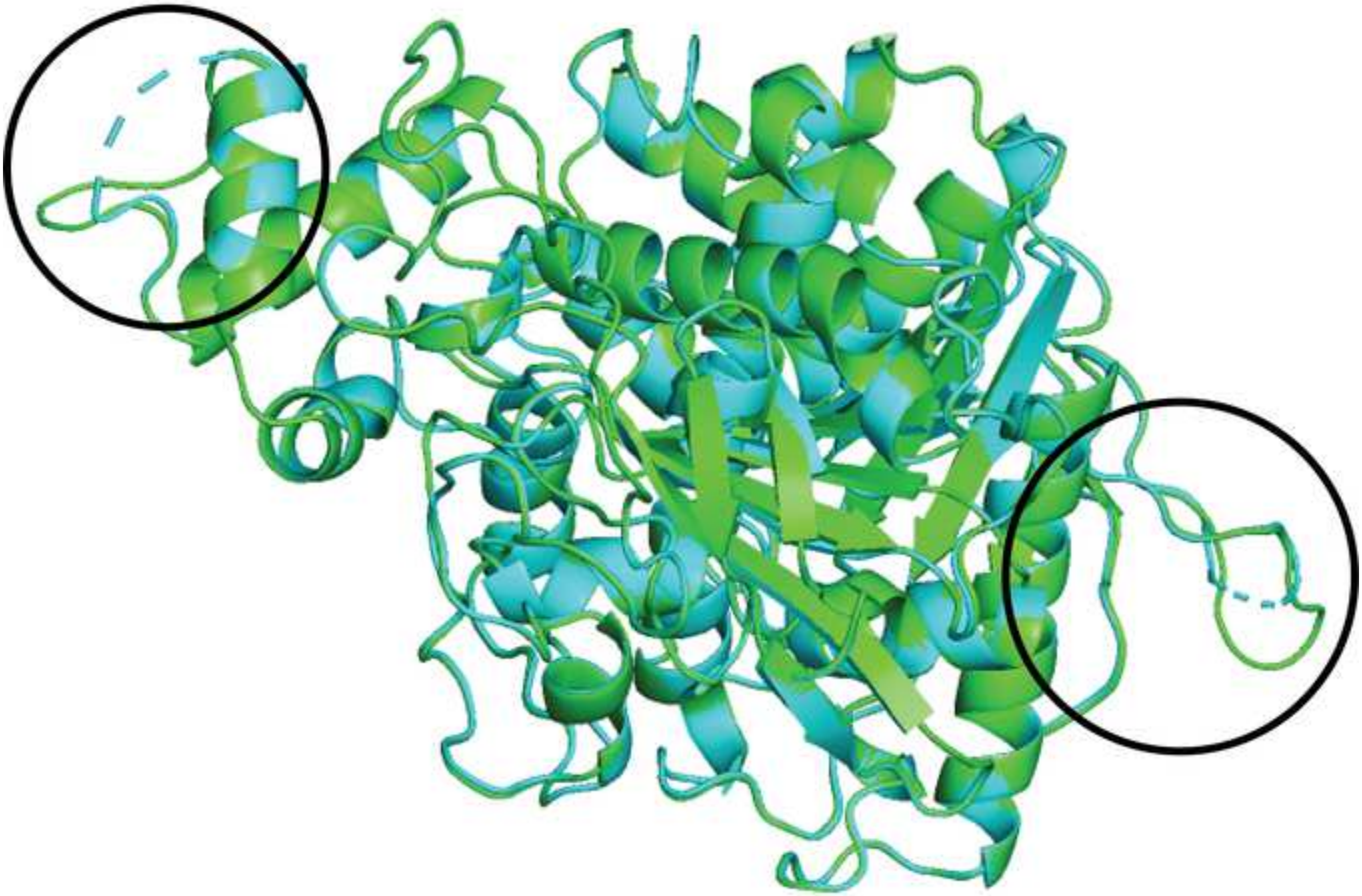

Figure 2

Click here to access/download;Figure;Figure 2.tif

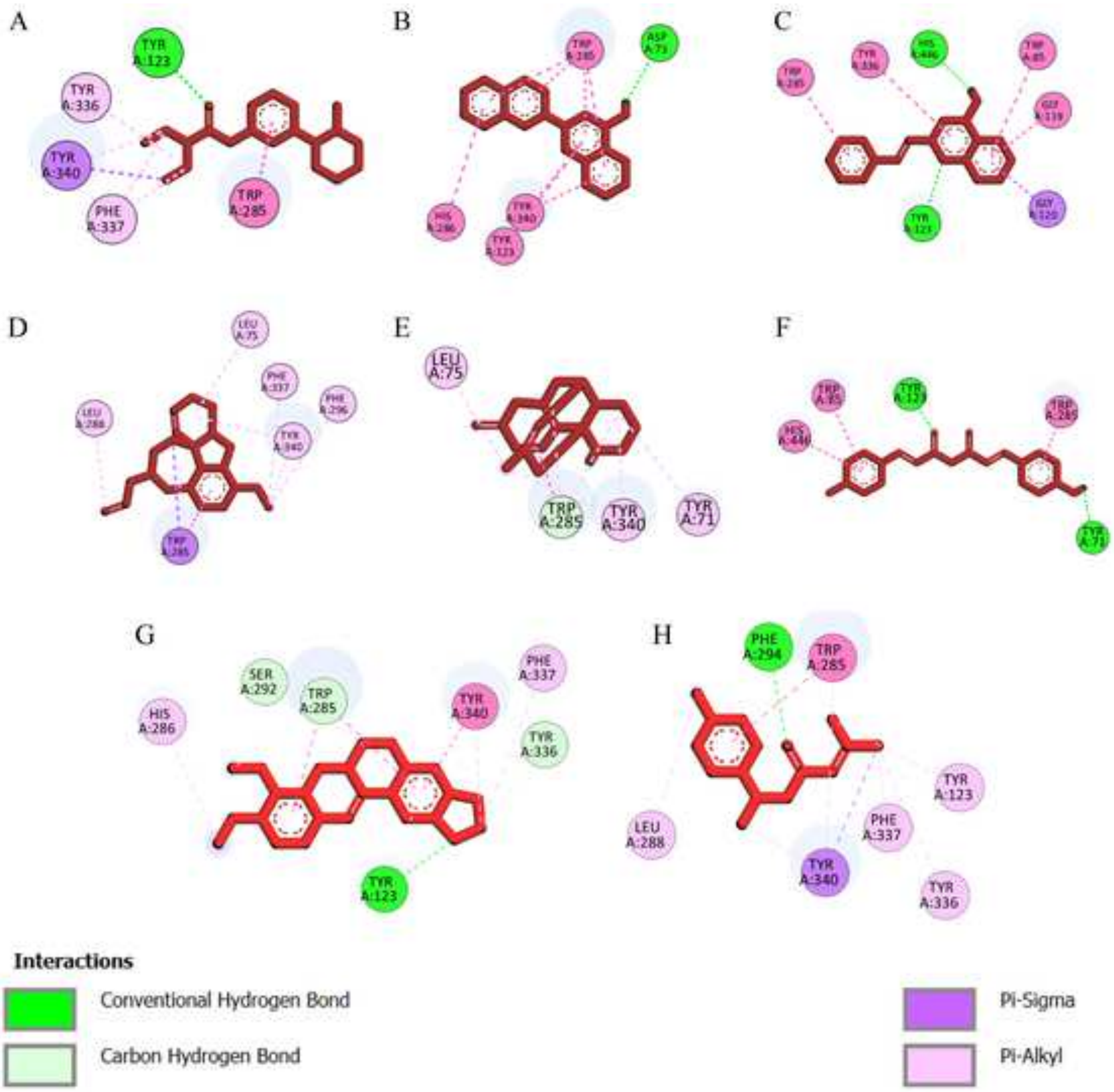

Figure 3

Click here to access/download;Figure;Figure 3.tif

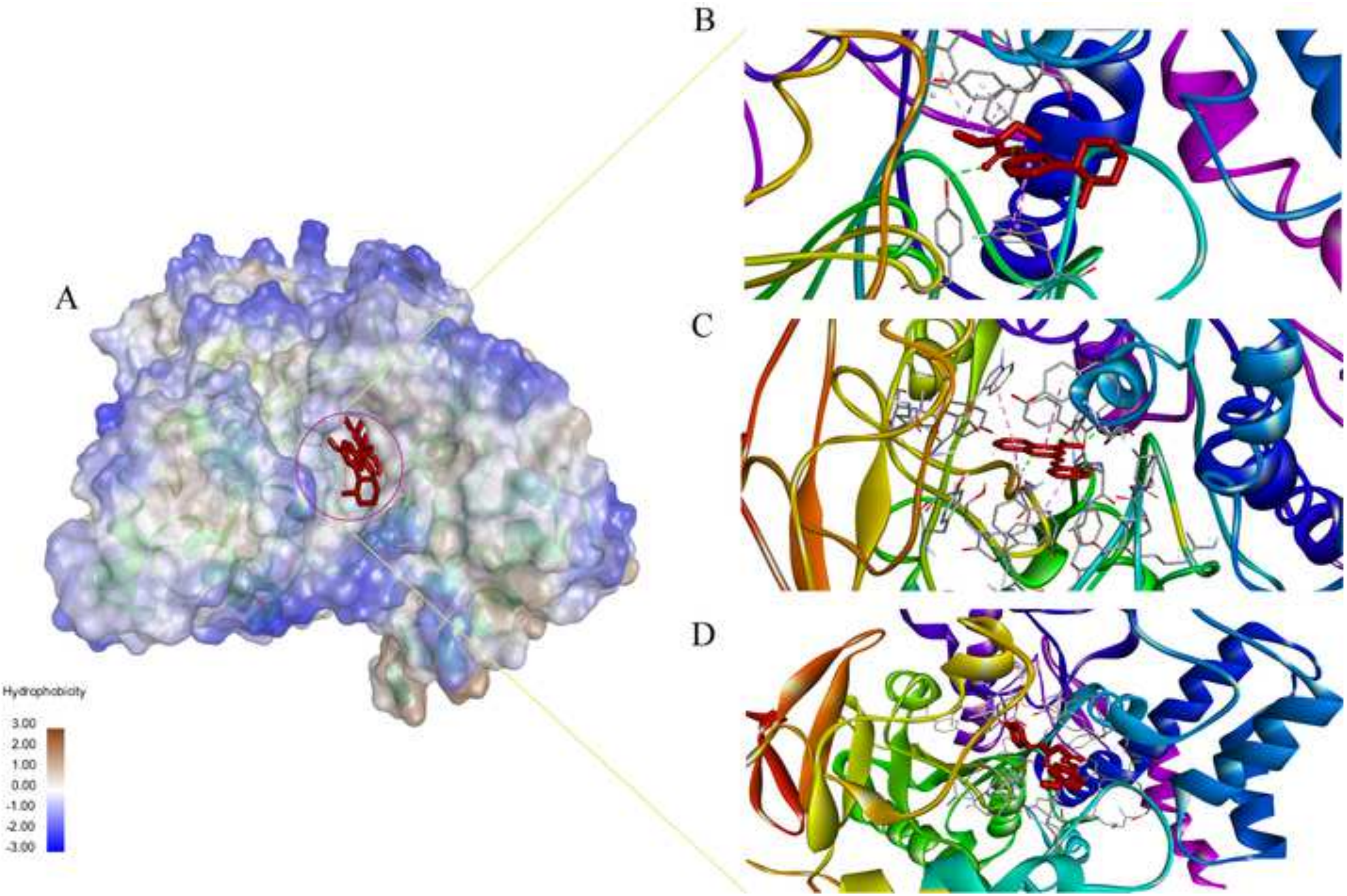

Figure 4

Click here to access/download;Figure;Figure 4.tif

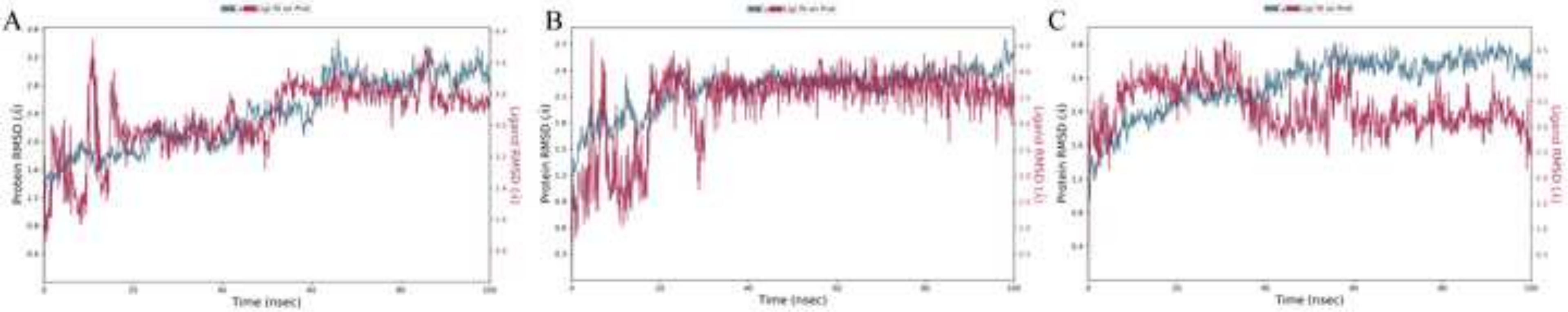

Figure 5

Click here to access/download;Figure;Figure 5.tif

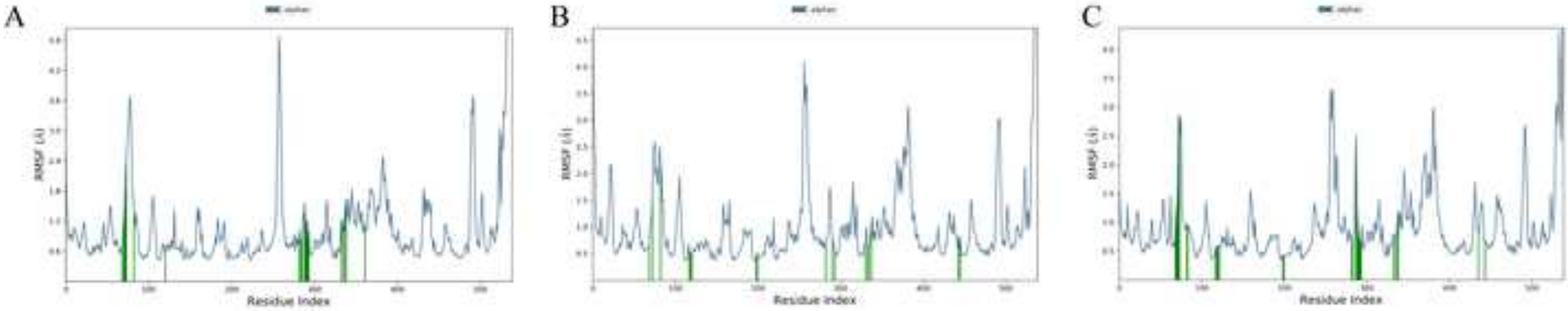

Figure 6

Click here to access/download;Figure;Figure 6.tif

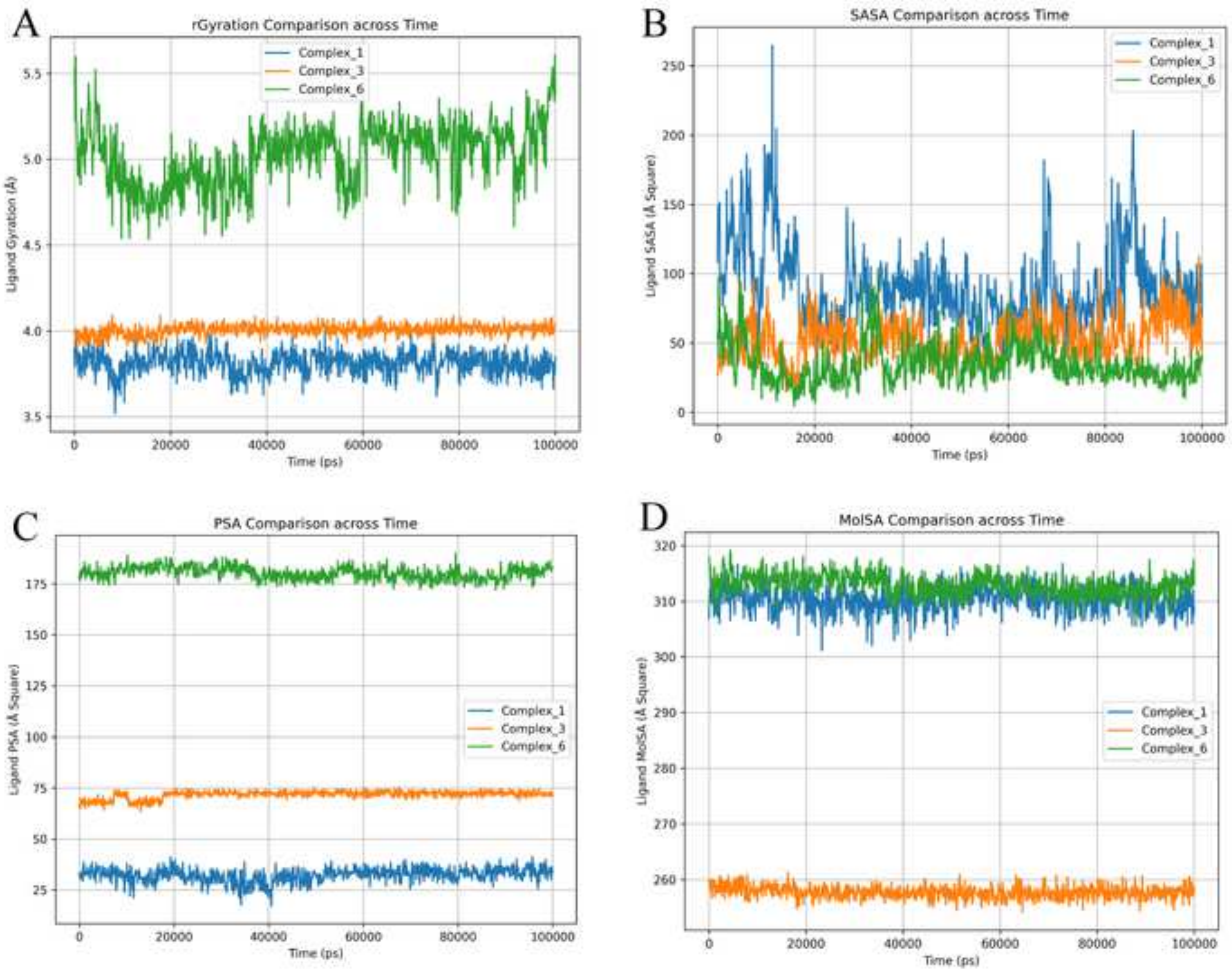

Figure 7

Click here to access/download;Figure;Figure 7.tif

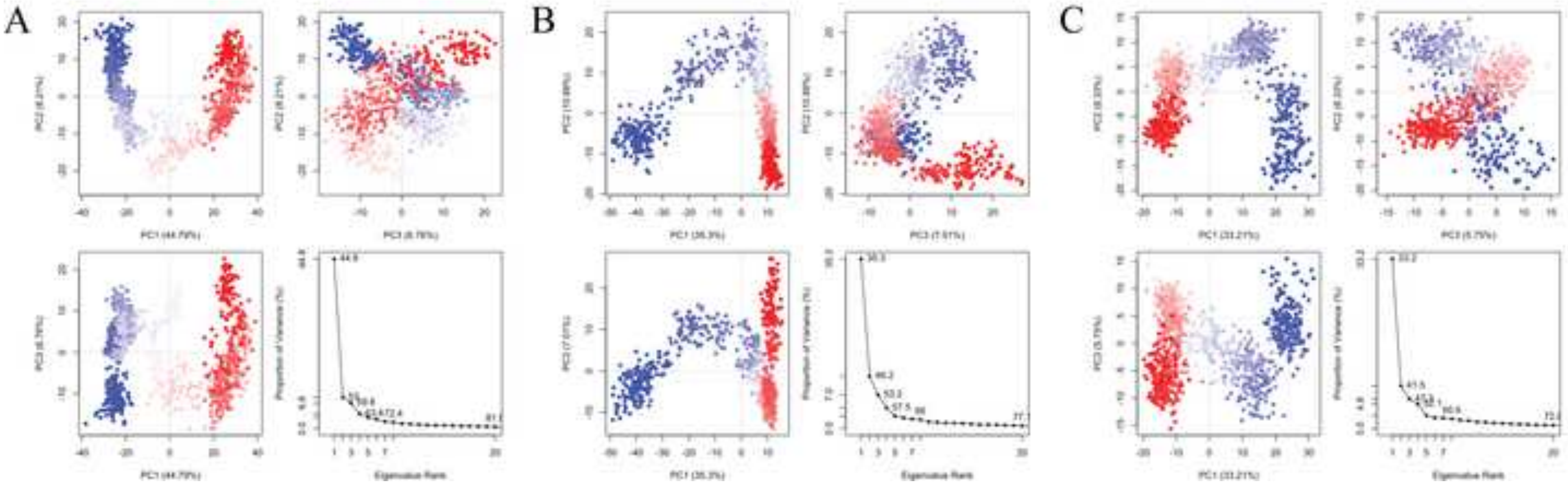

Figure 8

Click here to access/download;Figure;Figure 8.tif

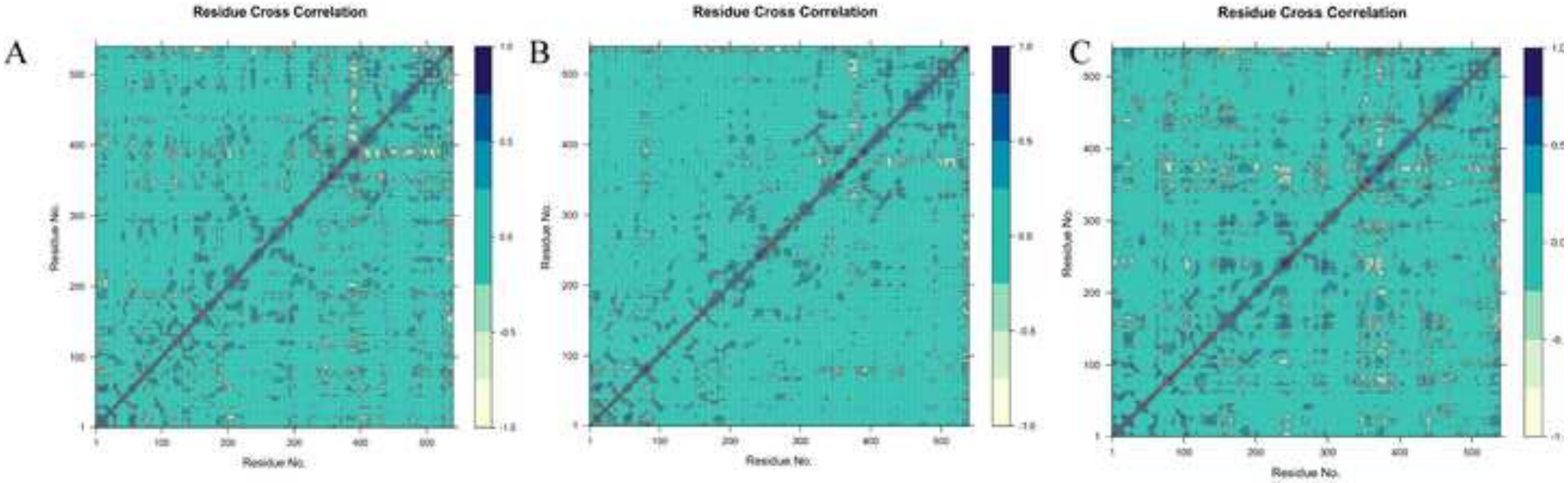

Supporting Information

Click here to access/download

Supporting Information

Supplementary Information.pdf
