# Supplementary material for: A computational approach to identify phytochemicals as potential inhibitor of acetylcholinesterase: Molecular docking, ADME profiling and molecular dynamics simulations
Source: PLoS One. 2024 Jun 4;19(6):e0304490. doi: 10.1371/journal.pone.0304490 (PMC11149856; doi:10.1371/journal.pone.0304490)
Supplement: Attachment — Submitted filename: Response letter.docx [file pone.0304490.s014.docx]

Response letter

Reviewer #1:

**Query 1.** A suitable comparison of acetylcholinesterase structure (original) and iTasser made (modelled) should be represented.

**Our response:** Thank you for your insightful suggestion, which will undoubtedly enhance the clarity and depth of the paper. In response to your query, we have recreated Figure 1 to facilitate a comprehensive comparison between the original structure of acetylcholinesterase (AChE) and the modeled structure generated by iTasser. To make sure the proper comparison of the Structure (original) and iTasser made (modelled) we make Figure 1 into three segments. The Crystal Structure of AChE Retrieved from RSCB-PDB in (A), The i-tasser Structure which was predicted (B), and the alignment between the RCSB PDB structure and the 3D predicted structure of the 4M0E protein is merged in (C) with all the missing residue 3 Letter ID and residues number.

Please check the revised figure 1 **“Figure 1. Structure of AChE.** (A) The Crystal Structure of AChE Retrieved from RSCB-PDB, (B) the I-tasser predicted structure, and (C) the alignment between the RCSB-PDB and predicted structures. The resolved missing residues and the conservation of the protein structure compared to its actual PDB sequence are shown.**”**

**Query 2.** Line no 458- in vitro and in vivo should be italicized.

**Our response:** We are deeply appreciative of the reviewer's discerning eye, as their attention to detail undoubtedly enhances the precision and integrity of our manuscript. We have italicized the terms '*in vitro*' and *'in vivo'* as requested, aligning with standard formatting conventions for scientific literature.

**Query 3.** The inclusion of MM/PBSA and MM/GBSA scores for the *In-silico* analysis is valuable. These methods provide computational estimates of binding free energy between a ligand and a receptor.

**Our response:** We sincerely appreciate the editors' insightful feedback, and it is undoubtedly an important analysis. These computational methods offer invaluable insights into the binding free energy between the ligand and receptor, enhancing the depth of our investigation. We have performed the MMGBSA for the best stable complex which was complex_1, complex_3 and complex_6. The data is added in tabular format in Table 8.

**Query 4.** Provide details about PCA and DCCM analysis, which software is being used and what are the parameters?

**Our response:** We thank the editors for this helpful comment. The Software and parameters were added in the method section of the revised manuscript. Please check the revised manuscript as “Analysis of PCA and DCCM were performed using Desmond software with default parameters (Malik et al., 2023; Rathod et al., 2023).”

**Query 5.** Use recent articles for your reference:- Jain, Mukul, Rupal Dhariwal, Krupanshi Bhardava, Sarvjeet Das, Mushtaque Shaikh, Reshma Tendulkar, Rashmi Wani, Meenakshi Sharma, Anil Kumar Delta, and Prashant Kaushik. "Insilico and invitro profiling of curcumin and its derivatives as a potent acetylcholinesterase inhibitor." Biocatalysis and Agricultural Biotechnology 56 (2024): 103022.

**Our response:** Thank you for your appreciation and acknowledgement of the significance of the article. We have indeed integrated insights from the recent work by Jain et al. (2024) to enhance our discussion on the phytochemical inhibitory analysis against our experimental protein, focusing particularly on the in-silico and invitro profiling of curcumin and its derivatives as potent acetylcholinesterase inhibitors.

**Query 6.** All the docking experiments should be run in triplicate and an average value should be given with a standard deviation. Apart from triplicate, 3 different docking software should be used.
**Our response:** We express our gratitude to the reviewer for their insightful comments. Conducting scientific experiments in triplicate format is crucial, and we have adhered to this standard practice. Docking was performed using PyRx and Autodock Vina in triplicate, with scores and standard deviations reported in the results section. Additionally, we utilized an online tool Hdock (<http://hdock.phys.hust.edu.cn/>) for the third analysis, and the data is available in the revised tables of the manuscript.

**Query 7.** As for molecular simulations, Desmond is being used but that also should run in triplicate to further validate the data.

**Our response:** We thank the reviewer for the comment. Molecular simulations using software follow certain computational algorithms and perform the analysis. In place of performing the analysis in triplicates using the same software, we have now used another software to reconfirm the findings of the simulation study.

Please check the revised methods section “Re-simulation for further validation of the data is performed by Gromacs simulation Software conserving the parameters unchanged. The stability was evaluated by comparing the root mean square deviation (RMSD), root mean square fluctuation (RMSF), and protein-ligand properties (radius of Gyration, SASA etc.).”

**Reviewer #2:**

**Query 1.** I appreciate the authors' efforts, but the most crucial point of this study is the molecular dynamics simulation part. Neither in the abstract nor in the conclusion does it include MDS data. This is the main thing! The fact that the MD results were good or showed good interactions was not an important sign that it would be a drug candidate, it could only be a suggestion!

**Our response:** We have checked the points raised by the reviewer, and are thankful for this insightful comment. The MDS data has been added in the abstract and conclusion section. Moreover, we have further improved our manuscript based on the suggestions from other reviewers. Please check the revised manuscript.

**Query 2.** The second most important omission is that the proposed phytochemicals as an inhibitor of AChE should have been discussed in the discussion section in terms of SAR and especially those with good MD and MDS results compared to standard drugs should have been compared with each other according to their similar structures and the skeletal structures and functional groups carried by the structure. Due to these general shortcomings, I inform you that publication in Plos One is not appropriate.

**Our response:** While I understand the importance of discussing the structure-activity relationship (SAR) and comparing phytochemical inhibitors with standard drugs based on MDS results, the scope of this paper primarily focused on presenting general trends and key findings. The methodology and results sections provided comprehensive data on the inhibitory activity of phytochemicals against AChE, laying the groundwork for future investigations. We believe that the current discussion adequately contextualizes the key findings of the study.

**Reviewer #3:**

**Query 1.** A Computational Approach to Identify Phytochemicals as an Inhibitor of Acetylcholinesterase: Molecular Docking, ADME profiling and Molecular Dynamics Simulations. It would be helpful to support the virtual conclusion using a simple in vitro assay for the most promising compound in your screening

**Our response:** We express our gratitude to the reviewer for their insightful comments. We also feel the necessity to validate the in silico results through wet lab experimentation. Due to a lack of wet lab facilitates and research funding, currently we aren’t able to make the in vitro experiment right. It would be an interesting thing to do in future.

**Reviewer #4:**

Overall, this is a very good research work integrating almost all-important computational tools. However, the authors need to address the following issues.
**Query 1.** May consider simulation for longer; may be up to **300 ns**, if possible.
**Our response:** We thank the reviewer for the comment. In the current situation, we are low on computational resources. That’s why we can’t extend the simulation up to 300ns. But, we have now used another software to reconfirm the findings of the simulation study.

**Query 2.** Must provide high-resolution images, especially Figures 4, 5, 7 and 8.
**Our response:** Thanks for the valuable comment, we have edited the Figures especially 4, 5, 7, and 8; and re-generate them. Please check the revised manuscript for the high-resolution images.

**Query 3.** Should be consistent in using abbreviations; define at first use.
**Our response:** Thanks for your valuable instruction. We have edited the abbreviations and rechecked by each of the co-authors.

**Query 4.** Be consistent in using the 'lower case' or 'upper case' first letter in the names of compounds or drugs.

**Our response:** Thankful to the reviewer for the insightful comment. We feel so sorry for these mistakes, the upper case and lower case are resolved and check further.
